# Aerosol Hygroscopicity and Surface-Active Coverage for the Droplet Growth of Aerosol Mixtures

**DOI:** 10.1021/acsestair.4c00303

**Published:** 2025-07-07

**Authors:** Nahin Ferdousi-Rokib, Kotiba A. Malek, Ian Mitchell, Laura M. Fierce, Akua A. Asa-Awuku

**Affiliations:** 1 Department of Chemical and Biomolecular Engineering, A. James Clark School of Engineering, University of Maryland, College Park, College Park, Maryland 20742, United States; 2 6865 Pacific Northwest National Laboratory, Richland, Washington 99354, United States; 3 Department of Chemistry and Biochemistry, University of Maryland, College Park, College Park, Maryland 20742, United States; 4 Department of Civil and Environmental Engineering, University of Maryland, College Park, College Park, Maryland 20742, United States

**Keywords:** aerosol hygroscopicity, surface-active organics, CCN, droplets, surface tension, carboxylic acids

## Abstract

The partitioning between inorganic salts and organic compounds within individual particles is a key factor that influences the uptake of water by particles. In this study, we investigated the aerosol hygroscopicity of ammonium sulfate (AS) and 2-methylglutaric acid (2-MGA) mixtures. 2-MGA is a moderately surface-active compound. Dilute surface tension measurements of 2-MGA/AS mixtures were taken by using a pendant drop goniometer. Hygroscopicity at subsaturated conditions was determined using a hygroscopicity tandem differential mobility analyzer (H-TDMA) and relative humidity was kept constant at 89 ± 0.9% RH. The droplet activation was also measured at supersaturated conditions using a cloud condensation nuclei counter (CCNC) from 0.4 to 1% supersaturation (SS). The single-hygroscopicity parameter κ was derived from measurements. Mixtures predominantly composed of AS, up to a 60 wt% 2-MGA, exhibit κ-values close to pure AS. However, κ decreases significantly as the organic fraction increases (>60 wt% 2-MGA). Previous predictions of κ-hygroscopicity assume full dissolution of both the organic and inorganic compounds. However, organic partitioning can influence the κ-hygroscopicity. A coverage-based parametrization, ϕ, assumes the probability of surface-active organics at the droplet surface. By estimation of the bulk and surface organic contribution, overall κ-hygroscopicity can be calculated. The model is computationally efficient, and the results indicate that organic solute depletion should be considered for fully soluble surface-active organics. Hygroscopicity predictions that account for the role of organic surface-active partitioning agree best with experimental results (*R* ^*2*^ > 0.95). Therefore, this study helps to enhance our understanding of cloud-forming properties of complex chemical mixtures containing surface-active organic and inorganic compounds.

## Graphical abstract

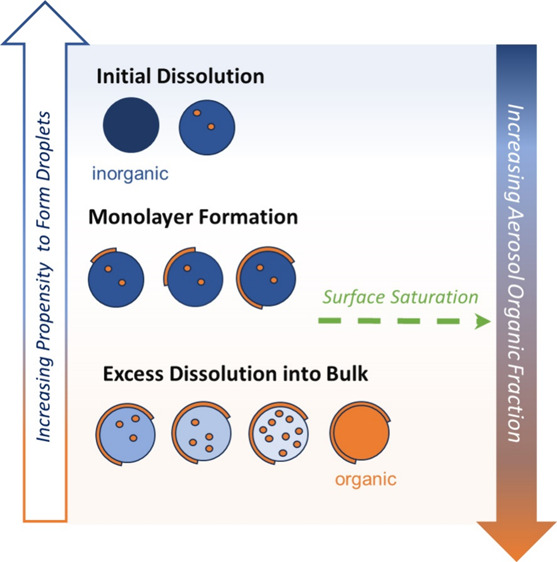

## 1 Introduction

Aerosol effects on the climate are a major source of uncertainty in model predictions of radiative forcing. Increases in the number concentration of aerosol particles modify the cloud reflectivity and lifetime. As a result, aerosols exert a strong, but uncertain, cooling effect on the climate.^1–3^ Aerosol particles are often mixtures of inorganic and organic components; their composition impacts their water uptake, hygroscopicity, and droplet formation. Cloud perturbations depend on the size and chemical composition of the aerosol particles that act as seeds for the cloud droplets. Previous field measurements have shown a major presence of internally mixed atmospheric aerosols containing both inorganic and organic components.^4–6^ Inorganic–organic aerosol mixtures may result in complex morphologies that modify aerosol surfaces and change droplet forming propensity. For example, an outer organic layer and inorganic core, referred to as liquid–liquid phase separation (LLPS) is known to modify droplet formation.^7–11^ Thus, aerosol chemical and physical compositions both influence subsequent particle water uptake and droplet activation.

Inorganic-containing aerosols are abundant in the atmosphere and are mainly composed of salts (e.g., AS). As a result, inorganic-containing aerosols have well defined water uptake (hygroscopicity) properties.^12–15^ However, organic-containing aerosols are more varied in composition and less understood. Organic-containing aerosols (OAs) constitute almost half of submicron-sized aerosols in the troposphere.^16–18^ OAs can react (age), oxidize, and form secondary OAs (SOAs) through various reaction pathways.^19^ Due to the evolving composition of OAs, changes in chemical reactivity, solubility, and phase transitions may occur, causing complexity in the understanding of their subsequent hygroscopicity.^20–23^

Previous studies have applied parametrizations of organic compounds, such as the oxygen-to-carbon (O/C) ratio, to improve water uptake predictions. Prior studies, such as Jimenez et al., have used O/C to identify organic-containing aerosol in field studies; O/C has subsequently been applied to estimate hygroscopicity (κ), suggesting a linear relationship between O/C and κ.^18,24–27^ Additionally, O/C has been applied to account for limited solubility and LLPS. Previously, Petters and Kreidenweis identified solubility limitations for hygroscopicity when compound solubility ranges from 0.1 to 100 g L^–1^.^28^ However, Nakao correlated solubility limitation effects for this range to organic compound O/C ratio >0.3, allowing the use of O/C ratio alone to estimate hygroscopicity.^26^ Previous literature shows that considering O/C limitations when estimating hygroscopicity may reproduce κ well, especially for CCN droplets.^9,26,27^

The uncertainty of aerosol water uptake is further complicated due to organic–inorganic interactions and their respective interactions with water on microscopic scales. To estimate the water uptake of aerosol mixtures, studies have compared experimental results to the hygroscopicity parameter predicted by *κ*-Köhler theory. Traditional Köhler theory assumes that all solutes present in the aerosol particle dissolve instantaneously into the aqueous phase after deliquescence. Complete and instantaneous dissolution is particularly true for inorganic-containing and OA with solubility greater than ∼100 g L^–1^.^28^ Furthermore, once dissolved in the bulk, the Zdanovskii–Stokes–Robinson (ZSR) mixing rule may be applied and assumes equal volume fraction contributions of individual components. If known, the partial solute water solubility can be directly applied in hygroscopicity models.^28,29^ If the composition is organic and unknown, the partial water solubility may be parametrized with its O/C ratio.^27,30^ In either case (whether the solubility is defined or parametrized), the thermodynamically ideal water-interaction assumptions of Köhler/ZSR are complicated by the presence of partially water-soluble compounds.^27,31–37^ For example, studies have shown that applying full dissolution assumption to slightly water-soluble to water-insoluble compounds results in an overestimation of *κ*-Köhler values compared to experimental measurements.^9,23,28,29^ It has been shown that for partially water-soluble to insoluble compounds, the fraction of compound dissolved in the aqueous phase must be accounted for in *κ*-estimations.^9,26,28,29,38^ Additionally, ZSR can be complicated by nonuniformly dissolved solutions and salting-out effects.^10,39,40^ In this paper, we focus on the inorganic-surface-active OA mixtures that are likely to readily dissolve in activated droplets but may present salting-out effects. Here, salting-out is defined as the propensity of inorganics to readily dissolve in the bulk water droplet and shift organic solute partition equilibrium from the bulk toward the surface of the droplet.^41,42^ Salting out has previously been attributed to enhancing surface tension depression; in particular, the presence of salt ions such as SO_4_^2–^ reduces the solubility of organic compounds due to its favorability to interact with water molecules present.^43–45^ As a result, salt ions in the presence of surface-active organics may further promote the organic partitioning behavior, which has been attributed to LLPS and enhanced surface tension depression over the droplet when the organic is surface-active.^43–47^

The above O/C and droplet activation studies do not account for the impact of the surface-active species. The presence of surface-active compounds is known to alter the surface tension of the droplet and will affect CCN activity.^48–52^ Surface-active organics partition to the gas/liquid interface of a droplet and results in an interfacial organic layer being formed.^50,51,53,54^ The presence of an organic layer at the surface modifies the vapor pressure across the droplet interface. The partitioning of organics to the surface may cause a change in droplet water activity; water activity is defined as the potential of a solvent to dissolve solute, at microscopic droplet sizes.^36,48,55–60^

Some studies have accounted for surface tension depression and CCN activity.^48,50–52,55,57,58,50–52,61–67^ Studies have proposed the use of predictive thermodynamic frameworks to account for surface-active organic partitioning and its influence on droplet surface tension. However, many of these studies that account for partitioning and phase separation on droplet growth use volume-additive assumptions despite the presence of distinct phases, especially those on the surface, which may drive droplet growth.^48–51,54,68,69^ Specifically, it has been shown that when an inorganic seed is coated with surface-active organic compounds, the organic partitions to the surface to form a monolayer.^50,51,54^ Surface tension depression is achieved when the monolayer reaches a minimum thickness.^49–51,70^ Previous studies have assumed a minimum monolayer thickness, ranging from 0.07 to 3 nm, and have modified surface monolayer thickness values to estimate droplet growth.^50,51,65,71,72^

The presence of a monolayer covering an aqueous inorganic core has previously been accounted for in estimations of the single-hygroscopicity parameter κ; however, models, such as the compressed-film model or LLPS models, have varied estimations of surface tension and κ.^50,51^ Additionally, the previously established models use concentration-dependent surface tension to estimate bulk concentration and water activity.^50,51,54^ Concentration-dependent surface tension models are effective but may also create challenges. The models are computationally expensive and may be difficult to integrate into larger-scale models (e.g., cloud parcel).^54^ As a result, current large-scale models do not account for the potential effects of surface-active organics on aerosol-cloud interactions. However, both surface tension and water uptake measurements may be used to develop a simpler framework to predict hygroscopicity without a surface tension solute concentration dependency. Compared to previous literature, we have performed surface tension, H-TDMA, and CCN analysis to estimate the probability of organic moving to the growing droplet air interface due to salting-out effects and the impact in predicting the κ-single-parameter hygroscopicity. To date, few binary mixed aerosol droplet studies have comprehensively evaluated sub- and supersaturated hygroscopicity, droplet surface tension, water solubility, and aerosol morphology simultaneously.

For this study, we study surface tension and hygroscopicity of AS/2-MGA aerosol mixtures. Althought it is only one component of many organics, 2-MGA, is considered one of the most abundant methyl-substituted carboxylic acids detected in atmospheric field studies.^73–75^ Additionally, 2-MGA is a known, soluble dicarboxylic acid that has exhibited a surface-active behavior; a previous study by Malek et al. estimated 2-MGA to be a representative of a moderately hygroscopic organic present in the atmosphere. However, the effect of surface activity on 2-MGA water uptake behavior has yet to be explored in binary aerosol mixtures with AS.^9^ Here, *κ* is first experimentally determined at sub- and supersaturated conditions and then compared with hygroscopicity prediction models. *κ* determined from experimental data employs equations and assumptions commonly applied to unknown aerosol composition and atmospheric aerosol studies.^76–82^ These values are compared to bottom-up hygroscopicity approaches that use parametrizations for full-solute dissolution, O/C, and organic surface tension depression by possible surface coverage. We subsequently discuss each method’s efficacy in predicting hygroscopic behavior for the 2-MGA/AS aerosol mixtures.

## 2 Experimental Methods

For this study, AS ((NH_4_)_2_SO_4_; Thermo Fisher Scientific, >99.0%) and 2-MGA (C_6_H_10_O_4_; sigma Aldrich, 98%) were used without further purification. A 0.1 g L^–1^ solution was prepared for each pure compound and binary mixture using ultra purified Millipore water (18 MΩ cm). Mixture compositions are provided in Table S1.

The surface tension of AS/2-MGA mixtures was measured using a pendant drop goniometer. The water uptake of the dry aerosol was measured using two experimental set ups. Polydisperse aerosols were generated by passing each solution through a constant-output Collison Nebulizer (Atomizer, TSI 3076). Wet aerosols were then dried (<5% RH) using two silica gel dryers in series. The H-TDMA measured droplet growth at subsaturated conditions (<100% RH) and a CCNC measured droplet activation in a supersaturated (>100% RH) environment. Both experimental setups are provided in Supplemental Figures S1 and S2.

### Surface Tension

Surface tension measurements for solutions containing 2-MGA, AS, and mixtures of both compounds were performed. To create the stock solutions, pure 2-MGA, a powder, was weighed out and dissolved in Millipore water; for mixtures, 2-MGA and AS were weighed out by mass for their respective mass ratios. Stock solutions were prepared at 1:5, 2:5, 1:1, 3:1, and 7:1 mass weight ratios of AS/2-MGA in 5 mL of Millipore ultrapure water and then diluted to concentrations as provided in Table S2. To observe the potential salting-out effects of AS, a notable salting-out agent, the dry mass of 2-MGA was kept constant in the stock solution and the dry mass of AS was increased. Mixture concentrations ranged from 0.002 to 0.013 M 2-MGA to represent diluted concentrations as an aerosol grows into a cloud droplet. Additional surface tension measurements of concentrated 2-MGA/AS mixtures were also taken. Concentrated mixtures were prepared similarly to dilute mixtures, with 2-MGA concentrations ranging from 2.053 to 10.264 M. All mixture compositions are reported in Supporting Information, Tables S2 and S3.

A pendant drop goniometer measured the surface tension at the droplet air interface (σ_s/a_) of the solution (Biolin Scientific Attention Theta Flex). Briefly described here, a mechanical microsyringe generated a droplet of the solution (<10 μL) at the tip of the needle. Images were taken at a rate of 60 frames per second until the values plateaued or the droplet fell from the needle. It is assumed that the surface tension reaches an equilibrium value with this measurement technique. Thus, σ_s/a_ (mN m^–1^) is determined from the fit of the droplet to the Young-Laplace equation.^83–85^ Subsequently, the average σ_s/a_ (mN m^–1^) from more than 100 images is calculated for all mixtures. The surface tension values were then averaged for each mixture. To characterize the surface activity of 2-MGA within the mixed droplets, σ_s/a_ is used to calculate the surface tension depression from that of pure water ( $\frac{-\Delta \sigma }{\sigma }=\frac{-\left(\sigma_{s/a}-\sigma_{w}\right)}{\sigma_{w}}$ ).

### H-TMDA

For H-TDMA measurements, the dry polydisperse aerosols were size selected at 100, 150, and 200 nm by an electrostatic classifier (DMA 1, TSI 3082; flow rate = 0.3 L min^–1^). The selected particles were humidified at 89 ± 0.9% RH using a Nafion humidification line (PermaPure M.H. series), and the size distribution was measured using a second electrostatic classifier (DMA 2, TSI 3082; flow rate = 0.3 L min^–1^). The H-TDMA experimental set up was calibrated using AS solution, with a *κ* value of 0.61.^86,87^ The ratio of the size-selected dry diameter (*D*_d_) and measured wet diameter (*D*_w_) equals the growth factor (*G*_F_). For calibration, dried AS aerosols were size selected at 100 and 150 nm. The aerosol *G*_F_ and relative humidity within H-TDMA were measured. Each calibration measurement was repeated five times, and *G*_F_ values are reported in Table S4. Growth factor is then used to compute the single-hygroscopicity parameter for subsaturated conditions, *κ*_H‑TDMA_.

### CCN

The aerosol water uptake properties were also measured in SS conditions (>100% RH) using a CCNC-100 (Droplet Measurement Technologies). The theory of the CCNC has been described in previous literature.^14,88,89^ The experimental measurement technique follows the protocol of Scanning Mobility CCN Analysis (SMCA)^90^ and is briefly described here. The dried polydisperse aerosols are first passed through an electrostatic classifier (TSI 3080) in scanning mode from 8 to 352 nm for 135 s. The aerosols are subsequently charged to facilitate size selection. The sheath to aerosol flow rate is 10 to 1 with an aerosol sample flow rate of 0.8 L min^–1^. The monodisperse size-selected particles were then sampled in parallel by a condensation particle counter (CPC, TSI 3776) and CCNC. The CPC operated at a flow rate of 0.3 L min^–1^. The CCNC operated at a flow rate of 0.5 L min^–1^. The CPC counted the number of dry particles (condensation nuclei, CN) at a given particle size. The particles are then also exposed to 0.4 to 1% SS within the CCNC, and the concentration of particles activated (CCN) was measured. The experimental set up was calibrated using AS.^14^ Calibration data are provided in Table S5 and Figure S3.

CCN data analysis of the calibration and all solutions were performed using Python-based CCN Analysis Toolkit (PyCAT 1.0); the open-source code is available on GitHub for public use.^91^ The activation ratio of CCN/CN was calculated for each dry particle size. A sigmoid was fit through the data to find the activation ratio and critical diameter (*D*_p,50_), where ∼50% of the dry particles form cloud droplets. It should be noted that a charge correction is applied in PyCAT.^92,93^ For each SS, critical diameters were found and used to calculate the supersaturated single-hygroscopicity parameter, κ_CCNC_.

## 3 Theory

Water uptake by internally mixed aerosol particles is often estimated using κ-Köhler theory,^36^ wherein the water activity of the droplet solution is parametrized using the hygroscopicity parameter *κ*. For this study, we quantify the role of phase partitioning in water uptake by comparing predictions of the traditional *κ*-Köhler model with (1) an O/C-solubility model and (2) a modified surface coverage model for a AS/2-MGA system.

### 3.1 Traditional κ-Köhler theory

Köhler theory describes the equilibrium water vapor saturation ratio at a droplet’s surface, *S*_eq_,^36,94^ which depends on its size and composition. This equilibrium relationship is a combination of the Kelvin effect, which describes the change in *S*_eq_ due to the curvature of the droplet, and the Raoult effect, which describes the change in *S*_eq_ due to the presence of a soluble substance in the droplet solution. The Kelvin effect is controlled by the surface tension at the droplet surface–air interface, σ_s/a_. The Raoult effect is controlled by the water activity of the droplet solution, *a*_w_. Combining both effects, the saturation ratio, *S*_eq_, over the droplet and the vapor pressure can be described as:

$$S_{\mathrm{eq}}=a_{w}\exp\left(\frac{4\sigma_{s/a}M_{w}}{\mathrm{RT}\rho_{w}D_{w}}\right)$$ 1

where *R* is the universal gas constant, *T* is the temperature, ρ_w_ is the density of water, *M*_w_ is the molecular weight of water, and *D*_w_ is the wet droplet diameter. Under the κ-Köhler model, *a*_w_ is parametrized as a function of the effective hygroscopicity parameter of the aerosol mixture, κ, assuming that aerosol components fully dissolve in water.^36,95^ The saturation ratio over the droplet is then given by:

$$S_{\mathrm{eq}}={\left(1+\kappa \frac{{D_{d}}^{3}}{{D_{w}}^{3}-{D_{d}}^{3}}\right)}^{-1}\exp\left(\frac{4\sigma_{s/a}M_{w}}{\mathrm{RT}\rho_{w}D_{w}}\right)$$ 2

The κ parameter can also be intrinsically calculated for chemically known water-soluble aerosols as follows:^95^

$$\kappa_{\mathrm{int}}=\frac{\nu_{s}\rho_{s}M_{w}}{\rho_{w}M_{s}}$$ 3

where *D*_d_ is the dry aerosol diameter, ν_s_ is the van’t Hoff factor, ρ_s_ is the density, and *M*_s_ is the molecular weight of the solute. To estimate κ for aerosols composed of more than one compound, the ZSR assumption can be applied via the mixing rule:^36^

$$\kappa_{\mathrm{ZSR}}=\sum_{i}\varepsilon_{i}\kappa_{i}$$ 4

where ε_*i*_ is the volume fraction of the individual component, *i.*

κ can also be derived directly from experimental data. For subsaturated conditions, the growth factor of the aerosol particle is related to κ as follows:^96^

$$\kappa_{H-\mathrm{TDMA}}=\frac{\left(G_{F}^{3}-1\right)}{\frac{\mathrm{RH}}{\exp(\frac{4\sigma_{s/a}M_{w}}{\mathrm{RT}\rho_{w}D_{d}G_{F}})}}-G_{F}^{3}+1$$ 5

where RH is the relative humidity of the H-TDMA system as a decimal. For supersaturated conditions, κ_CCNC_ can be described as follows:^36^

$$\kappa_{\mathrm{CCNC}}=\frac{4{\left(\frac{4\sigma_{s/a}M_{w}}{\mathrm{RT}\rho_{w}}\right)}^{3}}{27D_{p,50}^{3}\ln^{2}\mathrm{SS}}$$ 6

It is important to note that in traditional κ*-*Köhler theory, σ_s/a_ is that of pure water (in eq[Disp-formula eq1]), and the volume fraction of the surface-active compound fully contributes to overall hygroscopicity (eq[Disp-formula eq4]). These assumptions are modified in subsequent models.

### 3.2 O/C and Solubility Model

If the aerosol composition is known, thermodynamic solubility values can be directly incorporated into the Köhler theory.^28,29^ However, the majority of the organic-containing aerosol composition is unknown, and parametrization of O/C allows us to extend the theory to unknown organic aerosols. Previous literature suggests that hygroscopicity can have a linear relationship with O/C ratio.^18,24,25^ Nakao showed that the parametrization of κ in terms of the O/C can be defined as:

$$\kappa_{\mathrm{org}}=\frac{M_{w}}{\rho_{w}}\frac{12+\frac{H}{C}+16\frac{O}{C}}{7+5\frac{H}{C}+4.15\frac{O}{C}}\frac{1}{n_{C}}\frac{1}{12+\frac{H}{C}+16\frac{O}{C}}$$ 7

H/C represents the hydrogen/carbon ratio, and *n*_C_ represents the number of carbon atoms.

Other studies have also stated that O/C can influence the solubility of the organic compound^26,30^ and therefore have an impact on the solute effects incorporated in Köhler theory.^28^ Models have also shown that compounds with solubility of 0.1–100 g L^–1^ have a predominant effect on CCN.^23,28^ Volume-based solubility, ζ*,* can be parametrized by the O/C ratio via eq[Disp-formula eq8]:^26,30^

$$\ln\zeta =20\left[{\left(\frac{O}{C}\right)}^{0.402}-1\right]$$ 8

The dissolved fraction of organic solute, *x*_*i*_, can account for limited solubility and be incorporated into the mixing rule with the following definitions:

$$x_{i}=\frac{\left(\frac{D_{p,c}}{D_{d}}-1\right)\zeta_{i}}{\varepsilon_{i}}$$ 9

$$H\left(x_{i}\right)=\{\begin{array}{c} x_{i},x_{i}<1\\ 1,x_{i}\geq 1 \end{array},$$ 10

where *D*_p,c_ is the predicted critical wet activation based on compound intrinsic properties (e.g., molecular weight, density) and *H*(*x*_*i*_) is the scaling factor based on the amount of solute dissolved in the bulk phase.^97^ As a result, overall hygroscopicity based on solubility, κ_sol_, can be defined as:

$$\kappa_{\mathrm{sol}}=\sum_{i}\varepsilon_{i}\kappa_{i}H(x_{i})$$ 11

where ε_*i*_ and κ_*i*_ are the soluble volume fraction and hygroscopicity of each respective compound within the aerosol sample. For this study, the solubility of AS and 2-MGA is 764 and 40.6 g L^–1^, respectively.^98^

The solubility model is a modification of the traditional Köhler theory. Again, the above equations do not account for the presence of surface-active compounds. 2-MGA is a dicarboxylic organic acid with polar and nonpolar properties; when present in microscopic volumes of water, these organics can also influence droplet surface tension.^73–75^ The following hygroscopicity model considers a modified Köhler theory that incorporates the surface tension depression of an organic surface-active compound at the droplet interface in addition to a depletion of the solute from the bulk.

### 3.3 Modified Surface Coverage (MSC) Hygroscopicity Model

Previous studies have accounted for the partitioning of surface-active organics between the surface and bulk as well as its influence the Kelvin and Raoult terms. However, these studies use predictive adsorption isotherms to determine partitioning.^51,54,55,57,66^ Depending on the adsorption isotherm applied, the surface tension and κ can be varied. For example, a previous comparative study by Vepsäläinen et al. observed varied surface tension and partitioning predictions based on the adsorption model chosen (e.g., Gibbs, compressed-film model, and monolayer model).^69,99^ This can result in various predictions of the hygroscopic growth. Previous literature has attributed hygroscopic growth to the bulk composition of the aerosol and surface tension to aerosol surface composition.^50,51,54–56,100^ The differences in quantifying surface-active organic bulk-surface partitioning within microscopic systems can be addressed by using experimental observations to better estimate surface tension and κ-hygroscopicity.^59,101^

To account for the influence of surface-active organics on the droplet surface tension and water activity, we applied the MSC model. Finite layers of organic molecules, referred to as monolayers, may form on droplet surfaces,^49–51,54,49–51,102–104^ and these surface coatings may suppress σ_s/a_, enhancing water uptake by the droplet. Previous studies have applied the lowest possible surface tension value for organic compounds to determine the effects of monolayers on hygroscopicity. For example, Ruehl et al. apply the critical micelle concentration (CMC) surface tension of several organic acids as σ_org_, and surface tension values range from 30 to 46 mN m^–1^. Similarly, Ovadnevaite et al. used the average CMC surface tension of several organic acids (30 mN m^–1^) as *σ*_org_. It is noted that even initial lower surface tension values could be applied in the aforementioned models; however, use of the previously cited values and lower surface tension estimates (derived from concentrations of the fully dissolved organic solute) can overestimate the contributions of the organic monolayer.^69^ However, in organic aerosol systems, complete monolayer surface coverage may not always be attainable on the nanoscale systems.

We apply the principles of the previous work that shows that the surface tension at the droplet is typically overestimated with models that rely on the total solute concentration.^59,68^ Surface tension is traditionally measured with bulk measurements (De Nuoy ring, ∼50 mL; Wilhelmy plate ∼2 mL; and pendant drop tensiometer ∼10 μL) while aerosols form on microscopic scales.^105,106,107^ Recent studies have focused on surfactant concentration influence on microscopic scale surface tension with optical tweezer methods.^59^ Bain et al. show that the surface-to-volume ratio of a droplet influences σ_s/a_ and that a higher concentration of solute in activated droplets is needed to reach the lowest achievable surface tension compared with measurements made at the macroscopic scale. To achieve monolayer coverage in Bain et al., surfactant concentration of <100 mM was required. Surface-active organics, including surfactants, require much larger solute concentrations at the surface to achieve enhanced surface tension depression when the droplet radius is large. Based on these findings, the surface tension of the droplet can be >40 mN m^–1^ higher than that of the original macroscopic solution and bulk measurement. In this study, we will use surface tension values of dilute concentrations measured in the bulk to estimate the probability that organic material will move to the surface of the droplet and the subsequent surface tension depression of atmospheric droplets with smaller surface area-to-volume ratio. Specifically, we provide measurements for pure 2-MGA and AS mixtures at room temperature at dilute concentrations (Table S7). Again, mixture concentrations ranged from 0.002 to 0.013 M 2-MGA. Thus, in the regime of dilute concentrations, as the fraction of organic, *x*_*i*_, increases, there is an overall shift from the average σ_w_ to σ_org_, which resembles the change in σ_s/a_ commonly observed with Szyszkowski-Langmuir curves at constant dilution (shown in Figure S7). Using the average values over a range of solute concentrations from dilute experimental data, σ_s/a_®, σ_w_®, and σ_org_®_,_, we calculate the average droplet surface tension as

$$\overset{®}{\sigma_{s/a}}=(1-\varphi )\overset{®}{\sigma_{w}}+\varphi \overset{®}{\sigma_{\mathrm{org}}}$$ 12

φ represents the modified probability of the organic solute moving to the surface under dilute conditions as the organic faction in the solute composition increases. We note that as φ reaches 1, maximum organic phase partitioning is achieved, such that the organic surface is saturated, and organic mass moves to the bulk. Here, we assume that the surface-active organic moves to the surface of the aerosol droplet. φ is a parametrization dependent on 2-MGA/AS weight ratios and based upon average surface tension data. The parameter is weakly concentration dependent.

The MSC hygroscopicity model (κ_cov_) determines hygroscopicity with three categories: (1) initial dissolution of organic, (2) organic film formation on the droplet surface, and (3) the excess dissolution of organic into the bulk (Figure 1). To do so, the bulk fraction of 2-MGA is evaluated based on φ and the mass (*m*) of the organic partitioned to surface. For all cases, it is assumed that the total mass of AS (*m* ^*t*^_AS_ *= m* ^*t*^_inorg_) is fully dissolved, such that the mass of AS dissolved in the bulk, *m* ^*b*^_inorg_ *= m* ^*t*^_AS_ *= m* ^*t*^_inorg_. In the first category, the initial condition occurs for φ ∼0 and the solute mixtures are assumed to contribute to the Raoult effect and not to the Kelvin effect. At this condition, the bulk mass of 2-MGA (organic) in the aqueous phase, *m* ^*b*^_org_, is equal to the total aerosol mass of 2-MGA (organic), *m* ^*t*^_org_. Furthermore, κ can be determined via eq[Disp-formula eq11].

**Figure 1:**
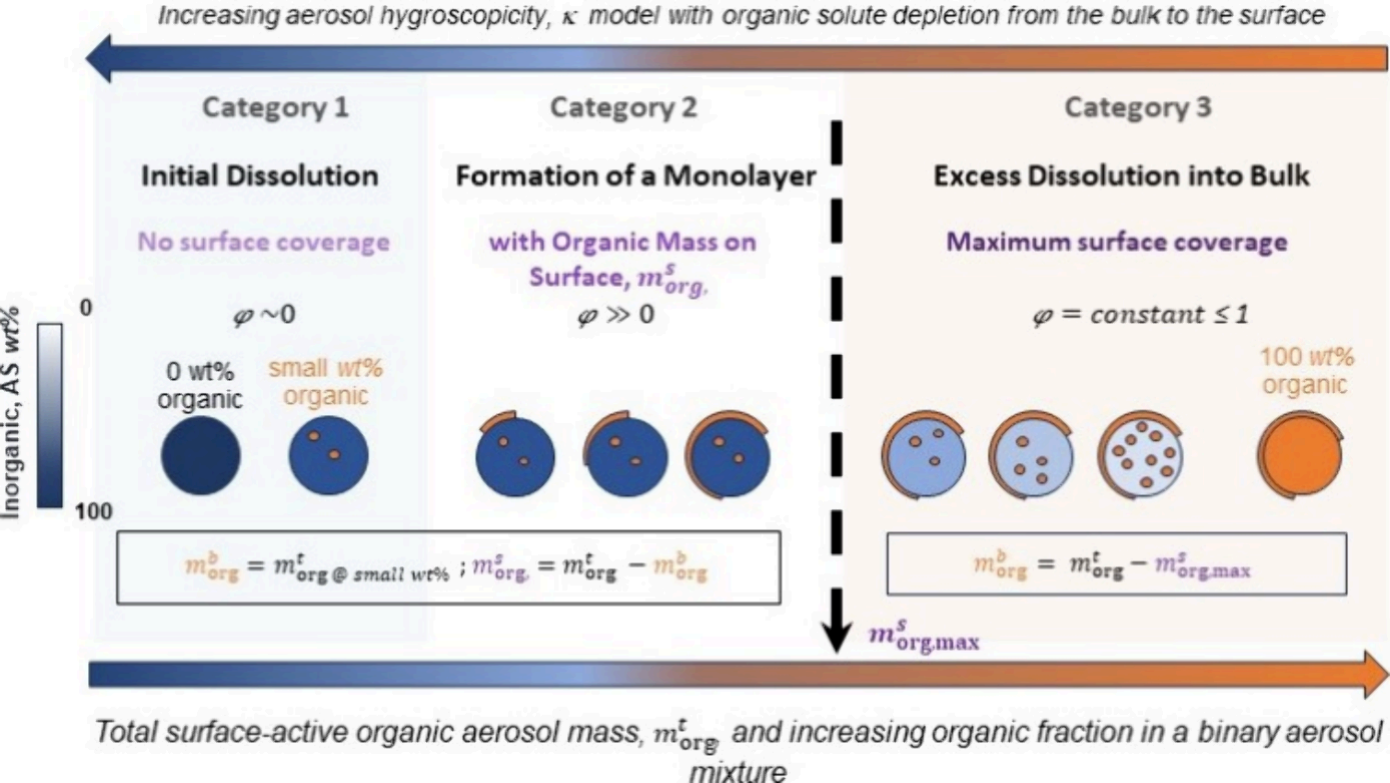
Visualization of the MSC model for an aqueous surface-active droplet growth process; AS is shown in blue, 2-MGA is in orange, and water is omnipresent in the bulk but not shown. The model considers an aerosol inorganic and an organic mixture. As the composition increases organic mass fraction (increasing orange, to the right), the decrease in overall hygroscopicity can be described by three categories 1, 2, and 3. The surface coverage probability parameter, φ, ranges from 0 to 1. Category 1 assumes no surface coverage, and both inorganic and organic solute dissolve in the droplet bulk solution. Category 2 assumes that additional organic mass moves to the droplet surface (*m* ^*s*^_org_). A maximum amount of organic solute moves to the surface (*m* ^*s*^_org,max_) as the aerosol organic fraction increases, and maximum possible surface coverage is achieved in category 3. In all cases, and even for a pure organic-containing aerosol, only the mass of the organic in the bulk (*m* ^*b*^_org_) and not at the surface contributes to the aerosol hygroscopicity. The proposed categorization and probability of movement of the organic to the surface are applicable to both sub- and supersaturated wet particles.

When the organic moves to the surface, a film could be present (φ somewhere between 0 and 1, category 2), and it is assumed that dissolution of 2-MGA into the bulk phase is halted. The concept of solute depletion during droplet formation has been previously postulated by Li et al. [107] and Sorjamaa et al. [55] but has yet to be applied to estimate the single-hygroscopicity parameter, *κ*. Therefore, in category 2, *m* ^*b*^_org_ is limited to a bulk mass where φ was formally ∼0. The remaining organic mass, *m* ^*s*^_org_, is assumed to partition to the surface and does not contribute to the hygroscopicity.

Eventually, σ_s/a_ of the droplet plateaus and organic surface coverage reaches a maximum and excess dissolution of the organic (category 3) occurs; thus, another critical point is reached where the mass on the surface reaches a maximum, *m* ^*s*^_org,max_. Definitions of each mass variable are listed in Supporting Information Table S6. The maximum is estimated at the onset of the surface tension depression plateau and as φ approaches 1. Any additional dissolution of the surface-active solute will contribute to the bulk concentration. Therefore, in category 3, the bulk organic mass in the droplet system can be estimated as follows:

$$m_{\mathrm{org}}^{b}=m_{\mathrm{org}}^{t}-m_{\mathrm{org},\max}^{s}$$ 13

A schematic of the model process and parameters of bulk organic mass for binary mixture hygroscopicity is shown in Figure 1. The organic and inorganic mass are then used to calculate a new volume fraction based on the bulk composition, ε_*i*_^*b*^, and the overall hygroscopicity, κ_cov_, is calculated as

$$\kappa_{\mathrm{cov}}=\sum_{i}\varepsilon_{i}^{b}\kappa_{i}H(x_{i})$$ 14

Eq. 14 can also be used to determine κ_cov_ values up to pure 2-MGA. All κ definitions are listed in Table S5.

## 4 Results and Discussion

### 4.1 Surface Tension

Pendant drop tensiometer measurements were taken for AS, 2-MGA, and 2-MGA/AS aqueous mixtures; measurements were performed by using a syringe needle to dispense and measure the curvature of droplets from stock solutions, as well as their dilutions. Surface tension experimental results are reported in Tables S7 and S8 and Figures S4–S7. For each stock solution, the dry mass of 2-MGA was kept constant, while the dry mass of AS was varied. Based on surface tension measurements at dilute concentrations of pure organic (∼0.002 to 0.01 M), 2-MGA is moderately surface-active. However, when the dry mass of AS is increased, surface tension depression is still observed up to a 1:7 2-MGA/AS solution, where surface tension becomes close to that of pure AS/water at (∼72 mN m^–1^) (Figure S7). Though the initial dry mass of 2-MGA is held constant, the presence of AS with 2-MGA lowers surface tension within the dilute mixtures; this can be attributed to AS being an effective salting-out agent and further promoting the surface partitioning of 2-MGA and depressing surface tension even within dilute concentration regimes.^44,45,47^

The partitioning of surface-active compounds and the resulting surface tension are dependent on the compound concentration.^48,64,67,69,104,108^ Previous studies have employed concentration-dependent surface tension data (e.g., Szyskowski-Langmuir, compressed-film model) to correct for hygroscopicity. For this parametrization, we use the average surface tension values of dilute concentrations to represent microscopic droplet atmospheric conditions and organic partitioning to the surface. To characterize the effect of 2-MGA on the solute mixture’s surface activity, the effective surface tension depression was calculated using measured average surface tension (Figure 1, Supporting Information Table S9). Surface tension depression is observed after 25 wt% 2-MGA. When the solute mixture becomes predominantly composed of the surface-active compounds (>60 wt% 2-MGA), surface tension depression plateaus. Figure 2a shows the average measured effective surface tension depression of dilute concentrations at the droplet interface for a given wt% of 2-MGA, shown in closed red triangles. In addition to the measured surface tension mixtures, the surface tension values were interpolated for mixtures that were generated via aerosolization (Figure 2a, open triangles). Surface tension values were interpolated from the surface tension trends observed between the measured surface tension values. The average surface tension values are then used to calculate the surface coverage probability parameter, φ, for binary aerosol mixtures via eq 12 (Figure 2b). Surface tension and average surface coverage probability parameters are used to define 2-MGA/AS mixtures into categories of possible surface coverage formation, shown in Figure 1, to inform the hygroscopicity parametrizations.

**Figure 2:**
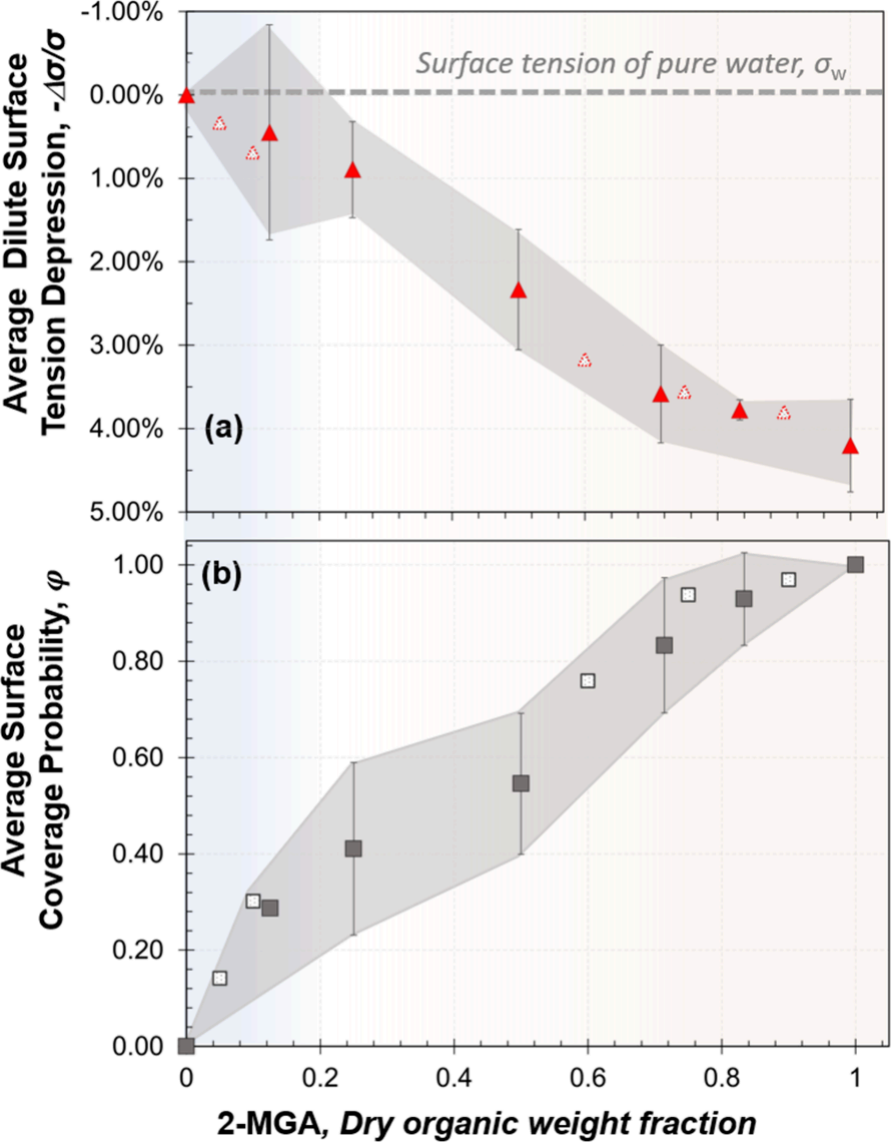
(a) Average measured surface tension depression (*−*Δσ*/*σ, triangles) as a function of 2-MGA organic wt% in a 2-MGA/AS mixture at 297 °K for dilute concentrations (red triangles) and interpolated mixtures (open triangles). When the organic mass fraction is zero, the measured σ is similar to that of pure water (σ_w,_ grey dashed line). (b) The estimated average surface coverage probability (ϕ) versus 2-MGA organic wt% in a 2-MGA/AS mixture for measured, dilute mixture (gray square) and interpolated mixture (open gray square). The parameter ϕ is derived from eq 12. The gray shaded region represents the range of values of measured surface tension and surface coverage probability parameter estimations in the dilute droplet bulk.

### 4.2 Organic Surface Coverage Probability Parameter, ϕ

The surface coverage probability parameter (*ϕ*) for each mixture was calculated by using eq 12 and is reported in Table S9 with average surface tension values. *ϕ* for experimental dilute mixtures (closed gray squares) and interpolated mixtures are shown in Figure 2b. Based on the surface tension measurements, we can estimate the bulk organic composition *m* ^*b*^_org_ as well as the probability of organic surface film formation of each mixture. Between 5 and 10 wt% 2-MGA, full dissolution of both the organic and inorganic occurs (solubility prevails over surface activity). This is category 1 and as a result, the surface tension in this range remains close to that of pure water, while *ϕ* is ∼0 (Figure 2a). After 10 wt% 2-MGA, the probability of organic surface coverage increases, corresponding to category 2 of droplet formation (partitioning). Figure 2b shows that for increasing wt% of 2-MGA, the likelihood of surface-active coverage is initially less than or equal to 20% and then plateaus after 60 wt%. From 25 to 60 wt% 2-MGA, a drop in surface tension corresponding with an increase in ϕ is shown (Figure 2b) and mixtures within this range are consistent with category 2 (Figure 1). Therefore, *m* ^*b*^_org_ = *m* ^*b*^_10 *wt%* 2‑MGA_ for mixtures between 25 and 60 wt%. After 60 wt% 2-MGA, a plateau in average surface tension depression and *ϕ* suggests that the organics will likely be found on the droplet surface. For the AS/2-MGA system, the maximum organic surface mass, *m* ^*s*^_org,max_, is determined at 60 wt% 2-MGA and applied to eq 13. This approach is then compared to experimentally and theoretically determined hygroscopicity values.

### 4.3 Sub and Supersaturated Hygroscopicity

Hygroscopicity of 2-MGA/AS mixtures was measured under both subsaturated and supersaturated conditions. Experimental *κ* *-*hygroscopicity for both H-TDMA and CCNC measurements was calculated using eqs 5 and 6, respectively. The experimentally derived hygroscopic values of both H-TDMA and CCNC measurements are shown in Figure 3; growth factor data from H-TDMA and activation diameter for each SS from CCNC measurements are given in Tables S9 and S10, respectively.

**Figure 3:**
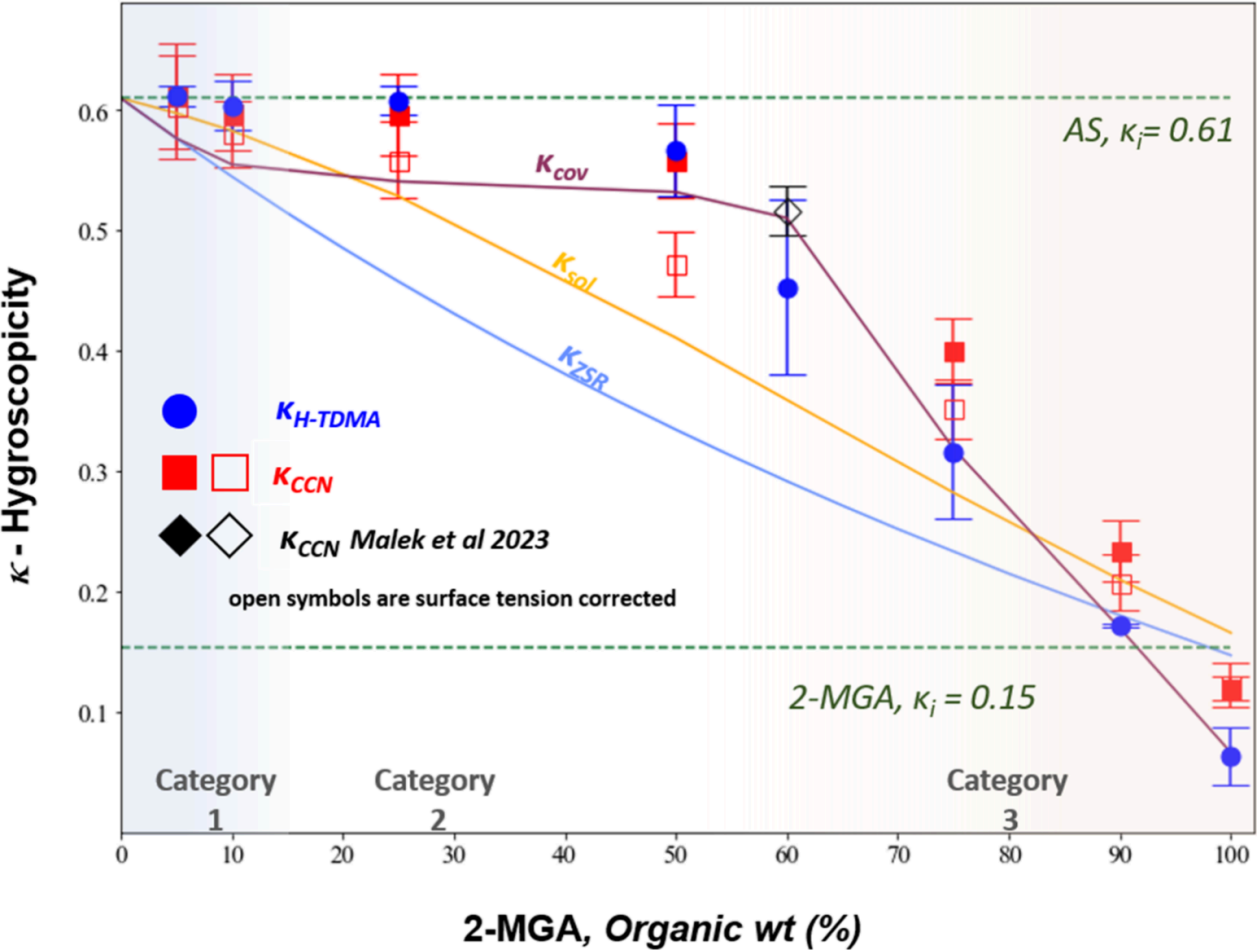
Experimental κ values derived from the CCN (closed red squares) and H-TDMA (closed blue circles) data sets are shown. κ_CCNC_ (closed red squares) is derived by assuming the surface tension of pure water in eq 6. κ_CCNC_ is also shown for a 60 wt% mixture (closed and open diamonds) obtained from data published in Malek et al.^9^ Open symbols are corrected with average surface tension data, as provided in Figure 2. The green dashed lines represent intrinsic κ-values of pure AS and pure 2-MGA, κ_int_ = 0.61 and κ_int_ = 0.15 (eq 3), respectively. Solid lines are predictions of the κ-hygroscopicity. The blue line, κ_ZSR_, is determined from eq 4 and is based on the traditional Köhler. The solid orange line is κ_sol_, from eqs 8–11 and employs a *κ* *-*hygroscopicity model as a function of solubility and O/C. The solid purple line, κ_cov_, is determined from eq 14 and accounts for surface activity and depletion of the organic from the bulk as visualized in Figure 1. Categories 1–3 of probable organic film formation at the droplet surface are labeled to indicate changes in the surface coverage model, as applied to the κ_cov_ hygroscopicity model.

For both H-TDMA- and CCNC-derived hygroscopicity values, aerosol mixtures between 0 and 50 wt% 2-MGA are similar to the intrinsic κ*-*value of pure AS, κ_int_ = 0.61 (Figure 3, eq 3). This suggests that for both κ_CCNC_ and κ_H‑TDMA_ below 50 wt% 2-MGA, AS is the main driver of aerosol hygroscopicity. When the aerosol mixture became predominantly composed of 2-MGA, κ_H‑TDMA_ decreases. From 50 to 60 wt% 2-MGA, a noticeable drop in κ_H‑TDMA_ from 0.53 to 0.44 is observed (Figure 3). Yet, at 60 wt% 2-MGA κ_CCNC_ remains close to pure AS, at 0.56. As aerosol mixtures become >60 wt% 2-MGA, hygroscopicity continues to decrease for both measurements, with κ_CCNC_ remaining higher than κ_H‑TDMA_. Both κ_CCNC_ and κ_H‑TDMA_ for the pure 2-MGA system are smaller than the intrinsically derived κ_int_ = 0.15, with κ_H‑TDMA_ significantly lower than 0.15.

κ*-*hygroscopicity calculated for supersaturated conditions typically assumes that the surface tension of the droplets is that of pure water. However, 2-MGA is moderately surface-active (Figure 2). To account for this difference, experimental κ_H‑TDMA_ and κ_CCNC_ was recalculated in eqs 5 and 6 using the average surface tension σ_s/a_® from diluted concentrations as a function of 2-MGA aerosol fraction (Figure 2 and Table S9). We refer to this adjustment in experimentally derived hygroscopicity values as κ_H‑TDMA‑ST_ and κ_CCNC‑ST_. Experimental subsaturated hygroscopicity effectively remains the same when accounting for surface tension (κ_H‑TDMA‑ST_ = κ_H‑TDMA_) (Table S10, thus not shown in Figure 3.). However, κ_CCNC‑ST_ is lower than κ_CCNC_ (Table S11); this can be attributed to surface tension (Kelvin) effects being stronger under supersaturated conditions compared to subsaturated conditions.^109^ The difference between κ_H‑TDMA_ and surface tension-modified κ_CCNC‑_ _ST_ is reduced.

The improved agreement between surface tension-modified κ_CCNC‑ST_ and κ_H‑TDMA_ suggests that an average surface tension approach based on dilute organic wt% composition (Figure 2) can improve κ*-*hygroscopicity estimates from supersaturated data; previous studies have attributed the difference between traditional κ_CCNC_ and κ_H‑TDMA_ to surface tension effects.^110,111^ To address these surface tension effects, previous studies have used concentration-dependent surface tension data to adjust water uptake predictions. However, in this work, we observe that parametrizing surface tension of dilute mixtures to account for the probability of organic at the droplet surface can also effectively reduce the gap between CCNC- and H-TDMA-derived experimental κ-hygroscopicity. Surface tension-modified experimental κ*-*hygroscopicity can subsequently be used to compare against the predictive hygroscopicity models (Table 1).

**Table 1:** Goodness of Fit (*R* ^*2*^) of κ-Hygroscopicity Models and Experimentally Derived κ-Values

κ model	*R*^*2*^ (κ_H‑TDMA_)	*R*^*2*^ (κ_CCNC‑ST_ ^a^)
κ_ZSR_—Köhler/ZSR	0.70	0.70
κ_sol_—O/C	0.71	0.83
κ_cov_—coverage-based	0.97	0.97

a Corrected with data from Figure 2.

Traditionally, Köhler/ZSR assumes full dissolution of the aerosol and droplet surface tension of σ_w_.^36,94^ Therefore, the Köhler/ZSR model estimates κ_ZSR_ as a volumetric weighted average of the respective solute κ_int_, as described in eqs 3 and 4. κ_ZSR_ (blue line, Figure 1) estimates a linear relationship between the hygroscopicity and organic mass fraction. The model has an *R* ^*2*^ value of 0.70 for both κ_H‑TDMA_ and κ_CCNC‑ST_. Though the model can estimate hygroscopicity for binary mixtures containing minimal organic (5–10 wt% 2-MGA), κ_ZSR_ underpredicts experimental κ-hygroscopicity from 25 to 75 wt% 2-MGA. For organic-rich mixtures (>75 wt% 2-MGA), κ_ZSR_ overpredicts subsaturated hygroscopicity. κ_ZSR_ also overpredicts pure 2-MGA as it utilizes κ_int_, derived from the known density and molecular weight of the organic compound. In short, assuming full dissolution and a droplet surface tension of σ_w_ fails to predict the binary mixtures’ initial plateau and then subsequent drop in overall hygroscopicity.

Previous studies have described the effect of solubility distribution within the bulk phase on hygroscopicity.^23,28,30^ For this study, 2-MGA is considered a soluble compound that may present limited solubility effects.^28^ Solubility effects were modeled using eqs 7–11 to determine the bulk fraction of 2-MGA and subsequent κ_sol_ as a function of O/C and solubility. Pure 2-MGA κ_org_ was calculated from O/C (eq 7) and estimated to be ∼0.17 (Figure 3) and higher than both experimentally κ_H‑TDMA_ and κ_CCNC‑ST_ values. The κ_sol_ model predicts full dissolution of the organic into the bulk using both water solubility of 2-MGA (40.6 g L^–1^) and O/C-solubility parametrization via eq 8. As a result, *H*(*x*_*i*_) (in eq 11) equals 1. Hence, κ_sol_ for the AS/2-MGA system is a similar hygroscopic trend as predicted by intrinsic κ and ZSR mixing rules using eqs 3 and 4. However, κ_sol_ differs from κ_ZSR_ because O/C is used to calculate 2-MGA κ_org_ (eq 7) as opposed to intrinsic properties (eq 3). κ_sol_ (orange line, Figure 3) has an *R* ^*2*^ value of 0.71 and 0.83 for κ_H‑TDMA_ and κ_CCNC‑ST_, respectively. The model is able to predict κ similar to experimental values of aerosol mixtures 5–25 wt% 2-MGA and 75–90 wt% 2-MGA, at the binary composition extremes; the model overpredicts hygroscopicity of pure 2-MGA. The solubility model poorly predicts κ for aerosol mixtures with 25–75 wt% 2-MGA. Again, the κ_sol_ model assumes that all organic mass dissolves in the bulk.^26^ Full dissolution does not adequately describe the hygroscopicity of this binary system nor can the oxidation state of the organic (O/C) describe the complex interactions and partitioning with the inorganic AS and water. The MSC model is subsequently applied to determine whether organic surface partitioning is a factor in the water uptake of this system.

The partitioning of surface-active organics may result in an organic solute at the droplet surface.^50,51,54^ The κ_cov_ model (purple line, Figure 3) uses the ϕ parameter (Figure 2) to determine the category to estimate the bulk composition. The MSC coverage-based model presents the best fit with an *R* ^*2*^ of 0.97 for both experimentally derived κ_CCNC‑ST_ and κ_H‑TDMA_. It is noted that for pure 2-MGA, the κ_cov_ model predicts the lowest value for the organic (κ = 0.06) yet is congruent with κ_H‑TDMA_ values for pure 2-MGA. Additionally, the κ_cov_ model estimates a minimal contribution of 2-MGA to the bulk phase in categories 1 and 2. For mixtures containing 5 to 10 wt% 2-MGA, it is predicted that 2-MGA fully dissolves into the bulk. The LLPS model introduced by Ovadnevaite et al.^51^ discusses the need for a minimum organic concentration in the droplet to form a monolayer. For mixtures up to 10 wt% 2-MGA, κ-hygroscopicity is lower than that of pure AS, demonstrating that water uptake is predominantly driven by AS but is also influenced by the presence of 2-MGA in the bulk. However, droplet surface tension remains close to pure water within this mixture range, which may be attributed to 2-MGA not having a significant influence on the Kelvin term due to a smaller probability that organics will not move to the droplet surface.^51^ Thus, we can approximate full dissolution of 2-MGA, similar to the traditional Köhler theory.

For mixtures >10 wt% 2-MGA and <75 wt% 2-MGA, the bulk composition of 2-MGA is held at the same concentration as the 10 wt% 2-MGA, with the remainder of 2-MGA partitioning to the surface. This trend is reflected by the surface tension depression within 2-MGA/AS mixtures (Figure 2) and is accounted for in the MSC model, where *m* ^*b*^_org_ = *m* ^*b*^_10 wt% 2‑MGA_ for mixtures <75 wt% 2-MGA. Organic solute will likely reach the surface of a droplet when the aerosol mass fractions of 2-MGA increase. Additionally, the presence of AS in the aqueous bulk can further drive out 2-MGA due to ionic behavior and stronger affinity to interact with water in comparison to the 2-MGA organic; this is referred to as the “salting-out” effect.^112^ Indeed, previous studies by Huang et al.,^113^ Malek et al.,^9^ and Ferdousi-Rokib et al.^114^ observed 2-MGA/AS LLPS aerosols where AS dominates the bulk and hygroscopic behavior. Furthermore, the partitioning and salting-out behavior leads to a reduced average depression in surface tension (Figure 2) and a roughly constant κ-hygroscopicity. This is because AS drives the hygroscopic behavior, while 2-MGA mainly modifies the Kelvin effect. Thus, accounting for this phenomenon by adjusting the bulk composition of the 2-MGA/AS aerosol droplets via the MSC model aptly mimics the plateau observed in experimental κ-hygroscopicity measurements from both CCN and H-TDMA. Moreover, the MSC model is an experimentally driven method that incorporates partitioning estimations not accounted for in the previous models. The use of intrinsic properties or molecular properties to estimate pure organic κ_org_, such as for traditional Köhler (κ_int_) and the O/C-solubility model (κ_org_), overepredicts organic hygroscopicity. This is a result of both models assuming that 2-MGA, a fully soluble organic, would behave as an ideal compound dictated by its molecular size and thermophysical properties.^115^ Surface tension and partitioning information derived from dilute surface-tension measurements improve the agreement between experimental and predicted organic hygroscopicity values.

In category 3, after the organic likely reaches the surface, the overall hygroscopicity drops due to the excess dissolution of the organic into the bulk. AS still remains fully in the bulk and promotes the salting out of 2-MGA, resulting in a surface tension depression. However, the aerosol droplet surface may reach a maximum organic concentration, which has previously been attributed to full monolayer formation.^50–52^ A previous study by Forestieri et al.^52^ observed a similar behavior in sea spray aerosols containing surfactants, where surface coverage reaches a maximum value. However, after a certain composition, additional organic was observed in the bulk, which was attributed to a monolayer collapse and dissolution of the organic into the bulk.^52^ Additionally, Petters and Petters found that limiting the number of surface monolayers to 1.5 for mixtures generally improves predictions of CCN activity for internally mixed aerosols containing surfactants.^65^ In the MSC model, we were able to reflect the point of dissolution at > 75 wt% 2-MGA, where surface tension depression plateaus and experimental κ starts linearly decreasing; this was done by adjusting bulk composition by holding *m* ^*s*^_org,max_ = *m* ^*s*^_60 *wt*% 2‑MGA_. By accounting for varied bulk compositions based on surface coverage probability and possible collapse, the κ_cov_ model best agreed with the trend observed in experimental κ. However, it is also noted that κ_cov_ aligns more closely with the experimental κ_CCNC‑ST_ than κ_H‑TDMA_ values, particularly at 60 wt% 2-MGA. This is likely due to the relationship of κ_cov_ with surface tension that has a more significant effect on CCN than H-TDMA. In addition, κ_cov_ also agrees with κ_H‑TDMA_ for the pure 2-MGA system and estimates the lowest predicted hygroscopicity for the pure system.

The MSC model can translate dilute average surface tension bulk measurements to a partitioning mechanism that effectively reflects the hygroscopic trend of 2-MGA/AS mixtures. Indeed, the concept of using surface tension data to parametrize water activity has been well established in previous studies.^50,54,116^ Previous studies predicting Kelvin and Raoult terms have based models on concentration-dependent surface tension data; however, the models result in various estimations of both terms. For example, Vepsäläinen et al.^69^ found varied predictions of both terms dependent on the adsorption model chosen, ranging from full partitioning for all mixture compositions of glutaric acid/AS to varied partitioning based on mixture composition.^69^ Similar to the previous studies, we have shown that integrating both salting-out and surface tension effects in a theoretical hygroscopicity model can affect water uptake predictions.^69,117^ However, we also demonstrate that average dilute surface tension data from pendant droplet measurements can also be effective in understanding salting-out/partitioning effects. This approach of exploiting dilute surface tension data to model organic partitioning in droplet growth is consistent with the recently published work of Mikhailov et al.^118^ who explore AS and D-glucose nanoparticles.^118^ By using an approach that does not depend fully on the concentration, we were able to simplify and improve water uptake predictions of surface-active 2-MGA/AS mixtures.

The use of average surface tension trends can be effective in replicating experimental water uptake trends, while also reducing the complexity. Ultimately, the framework introduced in this study relies on experimental data to inform the partitioning and water uptake processes for 2-MGA/AS mixed aerosols. The use of organic solute experimental data as opposed to κ values estimated from thermophysical properties (e.g., density, molecular weight, solubility, and molecular formula) can better reflect mixed aerosol water uptake. However, it should be noted that the MSC model deviates from the traditional concentration-dependent methodology that relies on ZSR volume additivity. It approximates the hygroscopicity from dissolved bulk mass and does not account for the surface film volume or number of monolayers. It also provides an approach to improve upon the single-hygroscopicity parameter in a more simple and quick manner. Indeed, the κ_cov_ *-*hygroscopicity model is not thermodynamically rigorous. Yet, like previous iterations of κ*-*hygroscopicity, κ the parameter seems capable of reflecting the hygroscopicity of mixed surface-active particles within the current uncertainties of measurements for mixtures.^36^ Moreover, as stated in Petters and Kreidenweis, the parametric solution is straightforward to implement in practice, computationally fast, and conceptually simple.^72^ Partitioning effects emphasized in the MSC model may be further enhanced with surface-active organics of lower solubility (e.g., adipic acid) and stronger surfactant strength. Previous studies have expressed the effect of surface tension being further complicated when the organic compound is limited in water solubility.^48,51^ Studies have incorporated solubility limitations into κ-predictions of partially water-soluble organic, insoluble organic, and its mixtures.^9,27,29,30,38,119^ However, further studies must be developed to determine the influence of surface activity in water-solubility limited mixtures. To efficiently predict κ-hygroscopicity for a surface-active organic/inorganic mixture, the predictive model must reflect the salting out and partitioning of organics, as shown by κ_cov_.

Ultimately, κ_cov_ is a modification of the widely used water uptake parametrization κ.^36^ However, previous studies have emphasized the correlation between particle size and CCN activation.^120–124^ For the 2-MGA/AS system in this study, one can consider the adjustment of organic solute (2-MGA) mass in the bulk as a modification of the effective particle size for AS activation. Here, 2-MGA bulk mass is attributed to surface-active partitioning; however, solute evaporation may also occur during water uptake measurements and contribute to bulk mass depletion.^125–127^ Thus, the loss of organic solute, whether through surface partitioning or to the gas phase via evaporation, would result in reduced CCN activation, even with the solute remaining in the solution containing a higher fraction of inorganic compounds. However, the hygroscopic properties of each solute component within the mixture remain constant; thus, the κ-parametrization is applied as a simplified approximation of water uptake; the κ_cov_ updated parameter is introduced to improve upon the water uptake approximation while accounting for partitioning. However, future work can explore the influence of evaporation-driven partitioning on κ and further improve upon this parametrization. Ultimately, improving *κ-*hygroscopicity, such as through the MSC model, is critical to advancing our understanding of CCN and droplet formation in aerosol-cloud systems.

## 5 Summary and Implications

The hygroscopicity of binary aerosol mixtures composed of AS, an inorganic salt, and 2-MGA, a surface-active organic, were investigated under subsaturated and supersaturated conditions. For subsaturated conditions, growth factors of size-selected particles at 89 ± 0.9% RH were measured. At supersaturated conditions, a CCNC measured and determined the activation ratio of particles from 0.4 to 1% SS. The hygroscopicity parameter, κ, was calculated for both conditions. In addition to hygroscopicity measurements, pendant drop tensiometer measurements of mixtures were taken at varying weight compositions and dilutions to obtain σ_s/a_ information. An average surface tension was calculated for each mixture as well as its corresponding probability of surface coverage parameter, ϕ. Experimental κ_CCNC_ and κ_H‑TDMA_ were adjusted by using average σ_s/a_ values to obtain κ_H‑TDMA‑ST_ and κ_CCNC‑ST._

Results show that for aerosols ≤50 wt% 2-MGA, both the H-TDMA and CCNC experimental κ-values agree. For all aerosol binary mixtures, the more water-soluble inorganic compound readily dissolves in the droplet. Surface-active water-soluble organics, such as 2-MGA, have the option of dissolving in the bulk or moving to the surface. For the system shown here, 2-MGA exhibits no water-solubility limitations and is estimated to contribute at least 10 wt% mass to the growing droplet. The remaining organic material was then moved to the droplet surface. This is congruent with a salting-out effect, where the organic dissolved material is removed from the bulk. Indeed, several atmospheric droplet papers have described this phenomenon including but not limited to refs 10,39,40,67 . These studies have described partitioning resulting in inorganic–organic-containing aerosol mixtures that undergo phase separation due to salting out. The process is attributed to AS having a high salting-out effect, reducing the activity of organic solute in the aqueous phase.^39^ In this work, we show that the salting-out phenomenon may lead to the formation of surface-active organics at the droplet surface that will modify the propensity of water uptake for aerosol mixtures.

Experimental κ-values were compared to two widely used and one modified κ-hygroscopicity theoretical model—traditional Köhler (κ_ZSR_), O/C-solubility (κ_sol_), and the MSC coverage-based models (κ_cov_), respectively. Both the traditional Köhler and the O/C-solubility model fail to predict the droplet formation of pure 2-MGA and the binary aerosol within the 25–75 wt% 2-MGA range. The organic mass can dominate ambient aerosols,^17,19^ and the 25–75 wt% 2-MGA organic–inorganic-containing aerosol mixture is ubiquitously measured in the ambient. Models such as κ_ZSR_ and κ_sol_ can inaccurately predict the overall propensity of organic–inorganic-containing aerosol mixtures to form droplets due to microphysical complexities, such as LLPS and surface activity; a compound which may be fully soluble, such as 2-MGA, may fully dissolve but as a result of salting-out effects and surface activity may instead partition.^9,48,66,68,69,99,128^ Thus, the intrinsic organic solubility is not an important parameter for the droplet growth process of the 2-MGA/AS binary system. Additionally, 2-MGA is one of the many organic compounds found in the atmosphere. Therefore, soluble surface-active organics, such as dicarboxylic acids, with potentially moderate hygroscopic behavior must be further studied to explore all of the effects of organic-droplet partitioning.

Ultimately, instantaneous, uniform, volume additive, and full dissolution assumptions of the inorganic–organic mixtures do not reproduce the experimental hygroscopicity results of the AS/2-MGA system. Instead, different categories of aerosol mixtures and droplet growth should be considered. Specifically, here, we account for moderately surface-active organics and the impact of surface activity on hygroscopicity with a surface tension coverage-based model. Experimental surface tension measurements were used to solve for the surface coverage probability parameter, ϕ, as a function of the fraction of organics in the aerosol. The model predicts the hygroscopicity of varying aerosol mixtures by sorting bulk-surface partitioning into three groups:

- Category 1: initial dissolution of organic (0–10 wt% 2-MGA)
- Category 2: movement of organic to the droplet surface and possible formation of a monolayer (25–60 wt% 2-MGA)
- Category 3: surface coverage at saturation—excess organic dissolves into bulk (>60 wt% 2-MGA)

The MSC model had the best fit (*R* ^*2*^ > 0.95) for both CCNC and H-TDMA hygroscopicity data and thus validated the proposed categorizations for droplet growth. The model demonstrates the ability to translate observed trends in dilute surface tension data to empirically constrain bulk and surface concentrations to predict κ-hygroscopicity. Furthermore, the results of this work show that we can use a simplified parametrization of partitioning effects to reflect experimental κ-hygroscopicity. Previous surface tension models have effectively used concentration-dependent parameters for water uptake; however, this study demonstrates that a generalized surface tension framework can also replicate the experimental κ-hygroscopicity of a surface-active organic/inorganic aerosol mixture. Furthermore, a simplistic approach such as the MSC model may be utilized by larger cloud models while being computationally less expensive. Although specific for the AS/2-MGA system, results may also be extended to other prevalent organic acid, weakly surface-active, and inorganic–organic mixtures in the atmosphere.

Ultimately, this work shows that the water uptake process of internal binary mixtures containing organic compounds can be modified by salting-out and surface tension effects. Indeed, the introduction of κ_cov_ in this work approximates the complex intermolecular interactions occurring between surface-active organics and inorganic. It is therefore critical to consider the evolution of surface and bulk droplet composition to predict hygroscopicity. The introduced κ_cov_ is a measurement-based framework that utilizes surface tension results to inform partitioning and water-uptake estimations. Thus, aerosol hygroscopicity and the readiness to form droplets is a measurable and predictable factor to account for nonideal thermodynamic solute–solvent interactions at microscopic scales. Future work should explore water-insoluble or partially water-soluble organic/surface-active mixtures to determine if any additive effects of organic surface tension and limited solubility exist. Additionally, the computationally efficient partitioning parametrization presented here should be tested within models of aerosol–cloud interactions, such as cloud parcel models or cloud-resolving regional or global models. Lastly, the κ_cov_ parametrization builds upon the size-independent water uptake parameter κ by accounting for surface-active partitioning. However, organic mass depletion may also occur due to other mechanisms, such as evaporation. Thus, future work should explore how organic evaporation may also influence the mixed κ-hygroscopicity. Ultimately, the results of this study will enable more complete simulation of complex mixtures of inorganic–organic aerosol that contain surface-active compounds, improving our treatment of regional haze and aerosol–cloud interactions.

## Supplementary Material



## Notes

Additional information on experimental mixture compositions, instrument set up schematics, definitions of water uptake parametrizations, experimental results for both surface tension and water uptake measurements (PDF)

## ‡

Department of Environmental Health and Engineering, Whiting School of Engineering, Johns Hopkins University, Baltimore, Maryland 21218, USA
