# Supplementary material for: Aerosol Hygroscopicity and Surface-Active Coverage for the Droplet Growth of Aerosol Mixtures
Source: ACS EST Air. 2025 Jul 7;2(8):1454–67. doi: 10.1021/acsestair.4c00303 (PMC12341549; doi:10.1021/acsestair.4c00303)
Supplement: Supplementary file 1 [file ea4c00303_si_001.pdf]

Supplemental Information:

Aerosol Hygroscopicity and Surface-Active Coverage for the Droplet Growth of Aerosol Mixtures

Nahin Ferdousi-Rokib[1^], Kotiba A. Malek[1], Ian Mitchell[1], Laura M. Fierce[2], Akua A. Asa-Awuku[1,3,4*]

[1]Department of Chemical and Biomolecular Engineering, A. James Clark School of Engineering, University of Maryland, College Park, College Park, MD 20742 USA

[2]Pacific Northwest National Laboratory, Richland, WA 99354 USA

[3] Department of Chemistry and Biochemistry, University of Maryland, College Park, College Park, MD 20742 USA

[4]Department of Civil and Environmental Engineering, University of Maryland, College Park, College Park, MD 20742 USA

[^]Now at Department of Environmental Health and Engineering, Whiting School of Engineering, Johns Hopkins University, Baltimore, MD 21218 USA

***Correspondence to:** Akua A. Asa-Awuku (asaawuku@umd.edu)

Contents:

I. Water Uptake Solution Composition
II. Surface Tension Solution Concentrations
III. Experimental set up for H-TDMA measurements
IV. Ammonium Sulfate calibration for H-TDMA
V. Cloud Condensation Nuclei Counter (CCNC) Experimental Setup
VI. κ Definitions
VII. Experimental Surface Tension Results
VIII. Summary of experimentally derived κ values

Supplemental Information

I. Water Uptake Solution Composition

Table S1. Mixture weight percentage and solute masses for water uptake measurements

Mixture (wt%)	2-MGA Mass (mg)	AS Mass (mg)
5% 2-MGA + 95% AS	1	19
10% 2-MGA + 90% AS	2	18
25% 2-MGA + 75% AS	5	15
50% 2-MGA + 50% AS	10	10
60% 2-MGA + 40% AS	12	8[a]
75% 2-MGA + 25% AS	15	5
90% 2-MGA + 10% AS	18	2
100% 2MGA	20	0

[a] Experimental result from Malek et al. 2023

II. Surface Tension Solution Concentrations

Table S2. Concentrations of dilute 2MGA/AS binary mixture for surface tension measurements

System	Water (mL)	2MGA (M)	AS (M)	AS (M)	AS (M)	AS (M)	AS (M)
1	5	0.014	1:5[$]<br>0.003	2:5[$]<br>0.006	1:1[$]<br>0.015	3:1[$]<br>0.045	7:1[$]<br>0.106
2[#]	8	0.009	0.002	0.004	0.009	0.028	0.066
3[#]	10	0.007	0.002	0.003	0.008	0.023	0.053
4[#]	20	0.003	0.001	0.002	0.004	0.011	0.026
5[#]	25	0.003	0.001	0.001	0.003	0.009	0.021

[$] Stock solutions were prepared at 1:5, 2:5, 1:1, 3:1 and 7:1 mass weight ratios of AS/2-MGA, the subsequent molar concentrations of AS are presented in the table.

[#]Stock solutions were prepared at 1:5, 2:5, 1:1, 3:1 and 7:1 mass weight ratios of AS/2-MGA in 5mL Millipore ultrapure water for System 1. Then System 2 through 5 are then generated by diluting concentrations of System 1.

Table S3. Concentrations of concentrated 2MGA/AS binary mixture for surface tension measurements

System	Water (mL)	2MGA (M)	AS (M)	AS (M)	AS (M)	AS (M)	AS (M)
6	2	2.053	0.076	0.757	1.514	2.270	
7	1	4.106	0.045	0.227	0.454		
8	1	5.474	0.061	0.605	3.027		
9	1	6.843	0.076	0.378	0.757		
10	0.5	8.211	0.303	3.027			
11	0.5	10.264	3.027				

III. Experimental set up for H-TDMA measurements

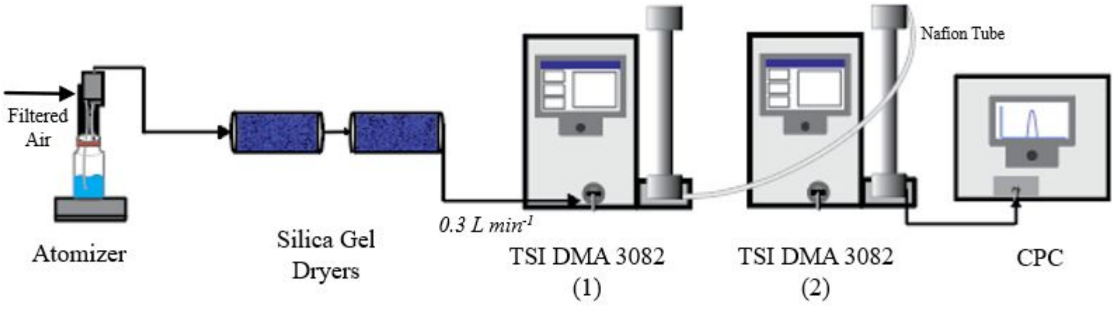

Figure S1. Experimental set up for H-TDMA measurements; dry, polydisperse aerosols were size selected through DMA1 at a 10:1 aerosol to sheath flow rate. The size selected particles are passed through a Nafion tube and then measured for growth factor using DMA2 and CPC.

IV. Ammonium Sulfate Calibration for H-TDMA

Table S4. H-TDMA Ammonium Sulfate Calibration

D_{set} (nm)	$D_{GeoMean}$ (nm)	G_F	RH
100	173.4	1.734	0.873
100	173.4	1.734	0.873
100	174.4	1.744	0.875
100	174.1	1.741	0.875
100	175.6	1.756	0.877
100	178.6	1.786	0.885
100	178.8	1.788	0.884
100	178.9	1.789	0.885
100	178.4	1.784	0.884
150	263.7	1.758	0.878
150	264.1	1.760	0.879
150	266.0	1.773	0.881
150	266.0	1.773	0.881
150	266.8	1.778	0.882
150	266.0	1.773	0.881
150	267.1	1.781	0.883
150	266.5	1.777	0.883
150	274.8	1.832	0.894
150	271.5	1.810	0.890
150	273.5	1.823	0.892
150	273.2	1.821	0.892
150	272.2	1.815	0.891

V. Cloud Condensation Nuclei Counter (CCNC) Experimental Setup

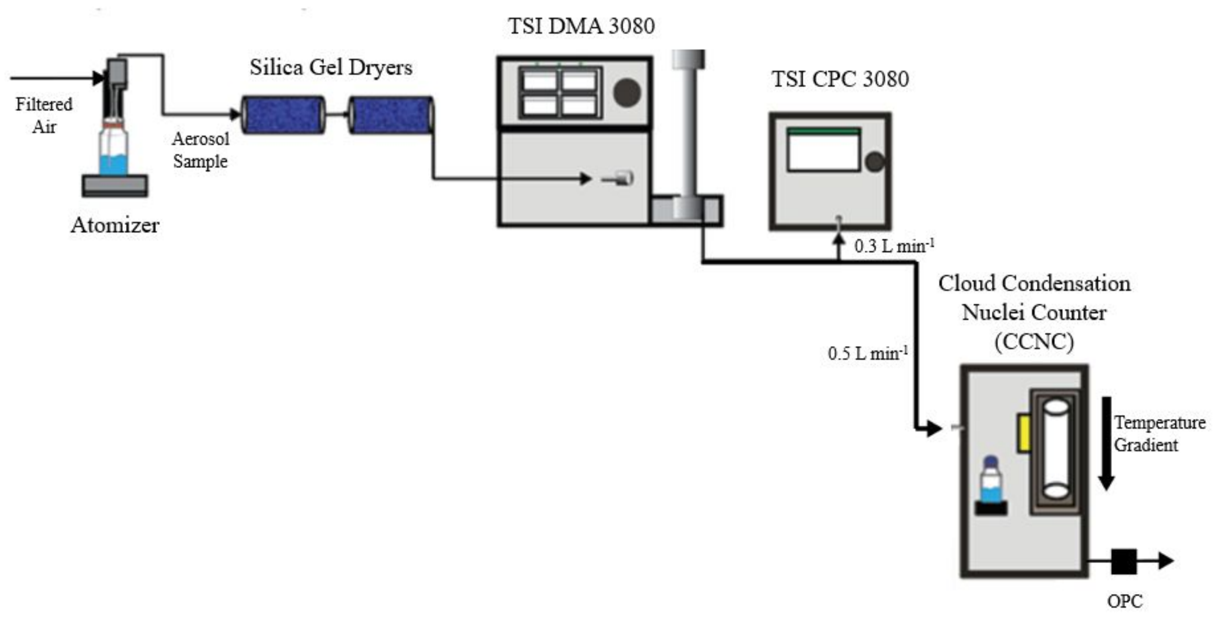

Figure S2. Experimental set up for Cloud Condensation Nuclei Counter (CCNC) experiments; dry, polydisperse aerosols were passed through the SMPS at a 10:1 aerosol to sheath flow rate; aerosols were flowed into the CPC and CCN at 0.3 L min^{-1} and 0.5 L min^{-1}, respectively.

Table S5. CCNC Ammonium Sulfate Calibration

Activation diameter (nm)	Calibrated supersaturation (%)
49.15	0.438
48.59	0.445
48.59	0.445
49.15	0.438
48.59	0.445
48.59	0.446
49.71	0.431
49.71	0.431
48.03	0.454
47.47	0.462
36.80	0.676
39.04	0.619
37.36	0.661
39.04	0.619
37.36	0.661
31.74	0.845
31.74	0.845
31.18	0.868
30.62	0.892
27.25	1.063
28.37	1.001
27.81	1.031
26.69	1.097
27.81	1.031
25.57	1.171
24.44	1

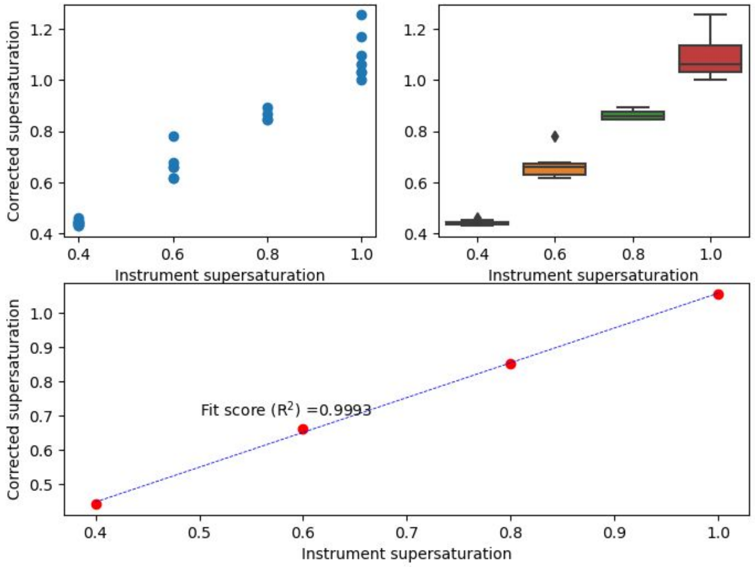

Figure S3. Ammonium sulfate (AS) CCNC instrument calibration

VI. Definitions

Table S6. Mass and Hygroscopicity Definitions for this study

Variable	Definition
$m^t{}_{\mathrm{i}}$	Total mass of solute (i = inorganic or organic) within the dry aerosol
$m^b{}_{\mathrm{i}}$	Mass of solute (i = inorganic or organic) dissolved in the aqueous aerosol bulk
$m^s{}_{org}$	Mass of organic solute partitioned to the surface
$m^s{}_{org,max}$	Maximum mass of organic solute partitioned resulting in organic monolayer saturation
$\kappa_{\mathrm{H\text{-}TDMA}}$	Experimental hygroscopicity based on subsaturated measurements and surface tension of water (72 mN m^{-1}) (Eq. 5)
κ_{CCNC}	Experimental hygroscopicity based on supersaturated measurements and surface tension of water (72 mN m^{-1}) (Eq. 6)
$\kappa_{\mathrm{H\text{-}TDMA\text{-}ST}}$	Experimental hygroscopicity based on subsaturated measurements and adjusted surface tension using surface tension measurements (Eq. 5 and 12)
$\kappa_{\mathrm{CCNC\text{-}ST}}$	Experimental hygroscopicity based on supersaturated measurements and adjusted surface tension using surface tension measurements (Eq. 6 and 12)
κ_{ZSR}	Predicted hygroscopicity based on traditional Köhler theory, using intrinsic properties to compute pure compound κ, assuming full dissolution, and surface tension of water (72 mN m^{-1}) (Eq. 3-4)
κ_{sol}	Predicted hygroscopicity based on O/C parameterization of solubility; pure organic κ is estimated from O/C (Eq. 7) and solubility limitations are estimated to adjust for fraction dissolved in the bulk (Eq. 9-11)
κ_{cov}	Predicted hygroscopicity based on adjusting bulk and surface organic mass using trends observed in surface tension measurements (Eq. 13-14)

VII. Experimental Surface Tension Results

Table S7. Surface Tension Results for Dilute 2-MGA/AS mixtures

AS (wt%)	2-MGA (wt%)	Water (mL)	AS (mol per kg H_2O)	2-MGA (mol per kg H_2O)	Surface Tension (mN m^{-1})	Std. Dev.
100.000	0.000	25	0.003	0.000	70.690	0.227
100.000	0.000	20	0.004	0.000	71.517	0.221
100.000	0.000	10	0.008	0.000	70.032	0.303
100.000	0.000	8	0.009	0.000	70.482	0.622
100.000	0.000	5	0.015	0.000	71.993	0.222
0.000	100.000	25	0.000	0.003	67.412	0.435
16.667	83.333	25	0.001	0.003	68.292	0.159
28.571	71.429	25	0.001	0.003	67.451	0.546
50.000	50.000	25	0.003	0.003	69.646	0.300
75.000	25.000	25	0.009	0.003	70.037	0.324
87.500	12.500	25	0.021	0.003	70.833	0.515
0.000	100.000	20	0.000	0.003	68.492	0.112
16.667	83.333	20	0.001	0.003	68.482	0.192
28.571	71.429	20	0.002	0.003	68.617	0.204
50.000	50.000	20	0.004	0.003	70.399	0.236
75.000	25.000	20	0.011	0.003	71.042	0.398
87.500	12.500	20	0.026	0.003	70.921	0.401
0.000	100.000	10	0.000	0.007	68.778	0.491
16.667	83.333	10	0.002	0.007	68.646	0.133
28.571	71.429	10	0.003	0.007	68.185	0.403
50.000	50.000	10	0.008	0.007	69.027	0.338
75.000	25.000	10	0.023	0.007	68.089	0.104
87.500	12.500	10	0.053	0.007	70.814	0.385
0.000	100.000	8	0.000	0.009	67.697	0.628
16.667	83.333	8	0.002	0.009	68.400	0.332
28.571	71.429	8	0.004	0.009	68.484	1.000
50.000	50.000	8	0.009	0.009	68.984	0.743
75.000	25.000	8	0.028	0.009	67.218	0.658
87.500	12.500	8	0.066	0.009	69.020	0.532
87.500	12.500	5	0.106	0.014	66.684	0.835
0.000	100.000	5	0.000	0.014	67.899	1.229
16.667	83.333	5	0.003	0.014	68.497	0.875
28.571	71.429	5	0.006	0.014	69.410	0.517
50.000	50.000	5	0.015	0.014	69.646	0.300
75.000	25.000	5	0.045	0.014	70.037	0.324

Table S8. Surface Tension Results for Concentrated 2-MGA/AS mixtures

AS (wt%)	2-MGA (wt%)	Water (mL)	AS (mol per kg H_2O)	2-MGA (mol per kg H_2O)	Surface Tension (mN m^{-1})	Std. Dev.
0.000	100.000	5	0.000	0.605	55.49	0.06
0.000	100.000	5	0.000	0.704	53.48	0.28
0.000	100.000	5	0.000	0.908	53.29	0.23
0.000	100.000	5	0.000	1.029	52.26	0.46
0.000	100.000	5	0.000	1.135	52.93	0.69
0.000	100.000	5	0.000	1.317	50.95	0.22
0.000	100.000	5	0.000	1.514	48.54	1.12
0.000	100.000	2	0.000	2.053	49.22	0.24
3.226	96.774	2	0.076	2.053	49.66	0.04
25.000	75.000	2	0.757	2.053	45.87	0.12
40.000	60.000	2	1.514	2.053	44.30	0.14
50.000	50.000	2	2.270	2.053	42.76	0.17
0.000	100.000	1	0.000	4.106	48.07	0.53
0.990	99.010	1	0.045	4.106	48.72	0.66
4.762	95.238	1	0.277	4.106	48.01	0.43
9.091	90.909	1	0.454	4.106	47.49	0.25
0.000	100.000	1	0.000	5.474	41.64	0.08
0.990	99.010	1	0.061	5.474	41.00	0.25
9.091	90.909	1	0.605	5.474	39.45	0.10
33.333	66.667	1	3.027	5.474	35.18	0.26
0.000	100.000	1	0.000	6.843	46.61	0.58
0.990	99.010	1	0.076	6.843	46.55	0.15
4.762	95.238	1	0.378	6.843	46.00	0.10
9.091	90.909	1	0.757	6.843	45.48	0.15
0.000	100.000	0.5	0.000	8.211	38.78	0.66
3.226	96.774	0.5	0.303	8.211	40.42	0.36
25.000	75.000	0.5	3.027	8.211	38.90	0.17
0.000	100.000	0.5	0.000	10.264	36.60	0.35
0.000	100.000	0.5	0.000	11.352	41.85	0.49
0.000	100.000	0.5	0.000	15.135	40.29	0.23

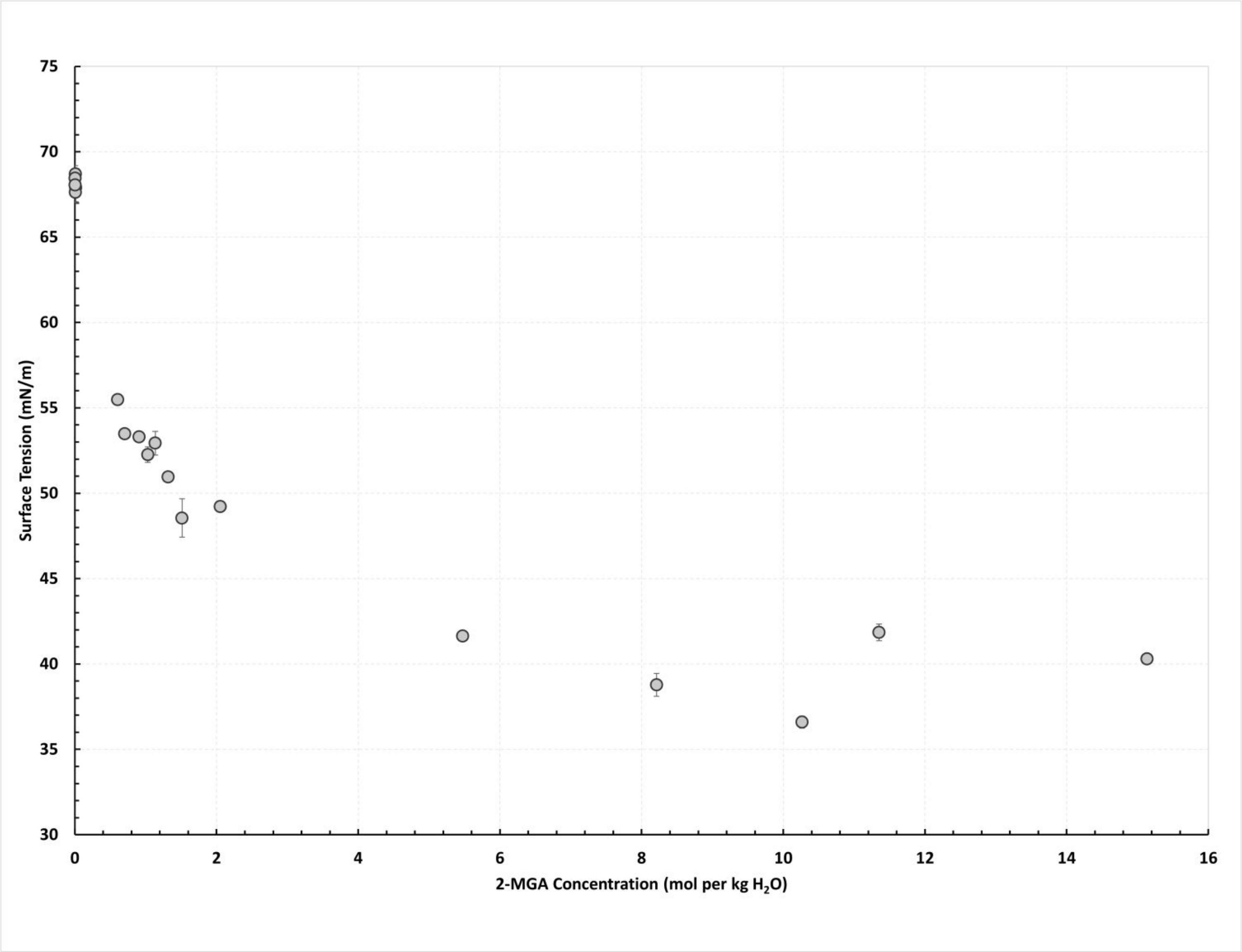

Figure S4. Surface tension values of pure 2-MGA/water solutions versus 2-MGA molarity

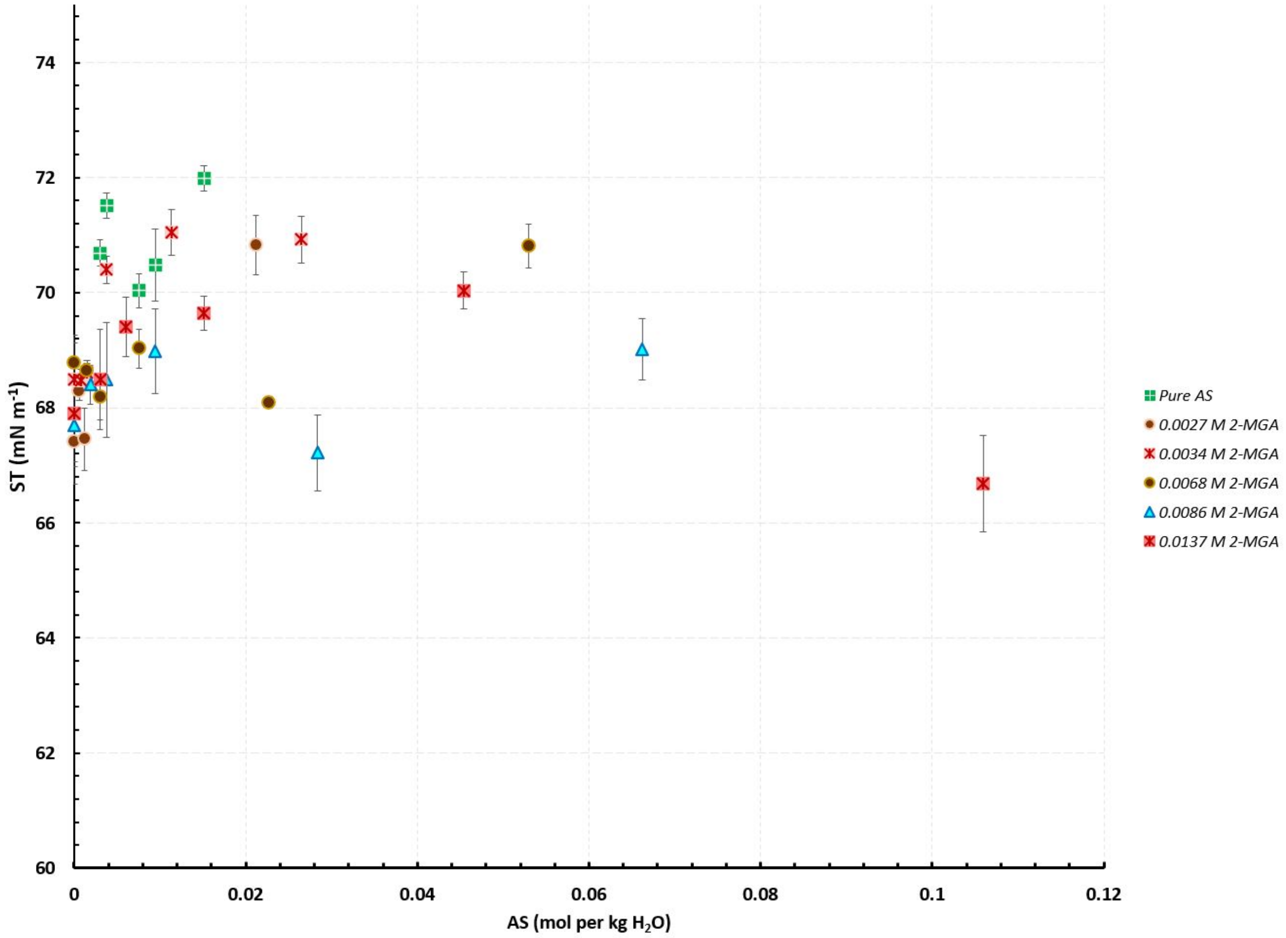

Figure S5. Surface tension values of dilute 2-MGA/AS/water solutions versus AS molarity

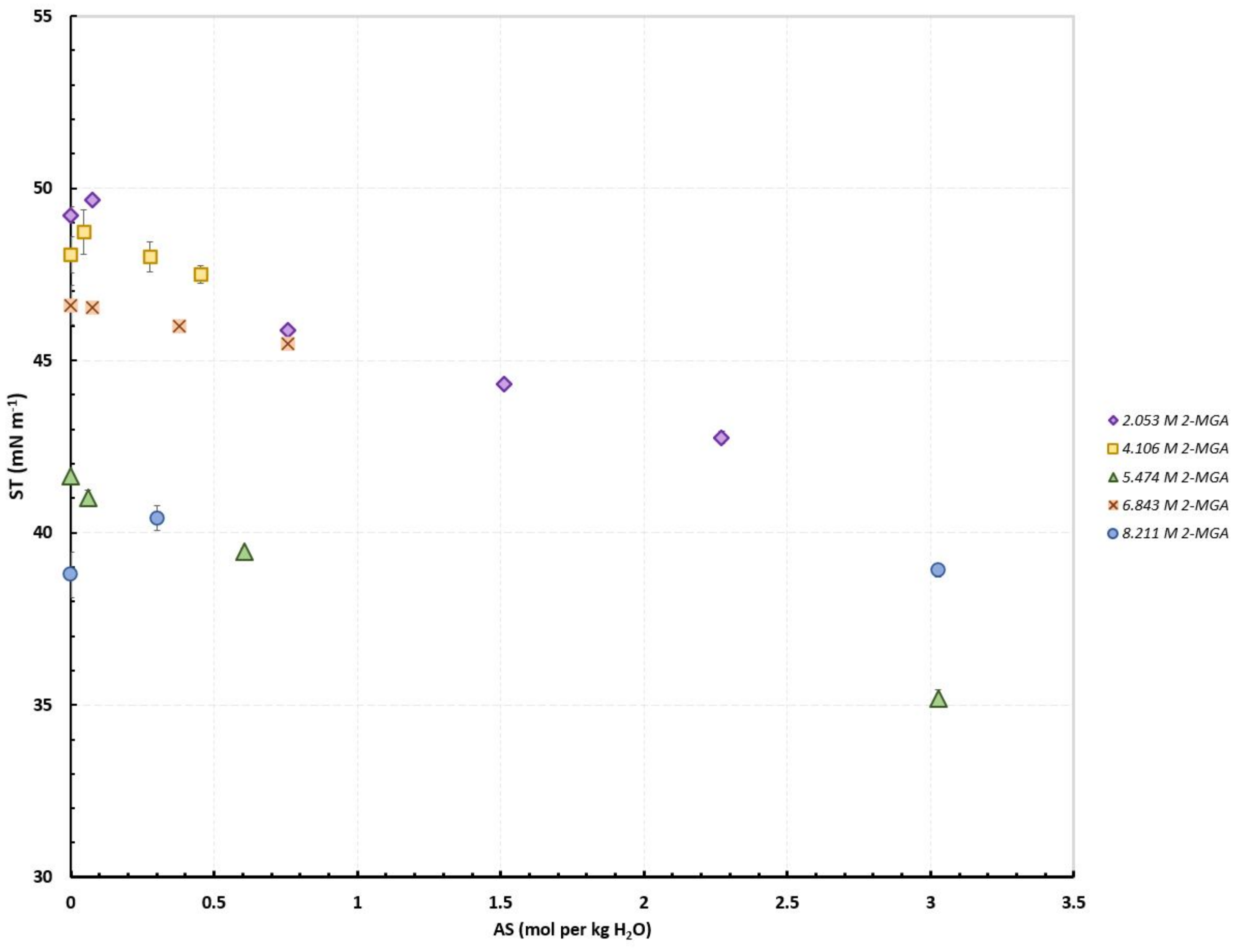

Figure S6. Surface tension values of concentrated 2-MGA/AS/water solutions versus AS molarity

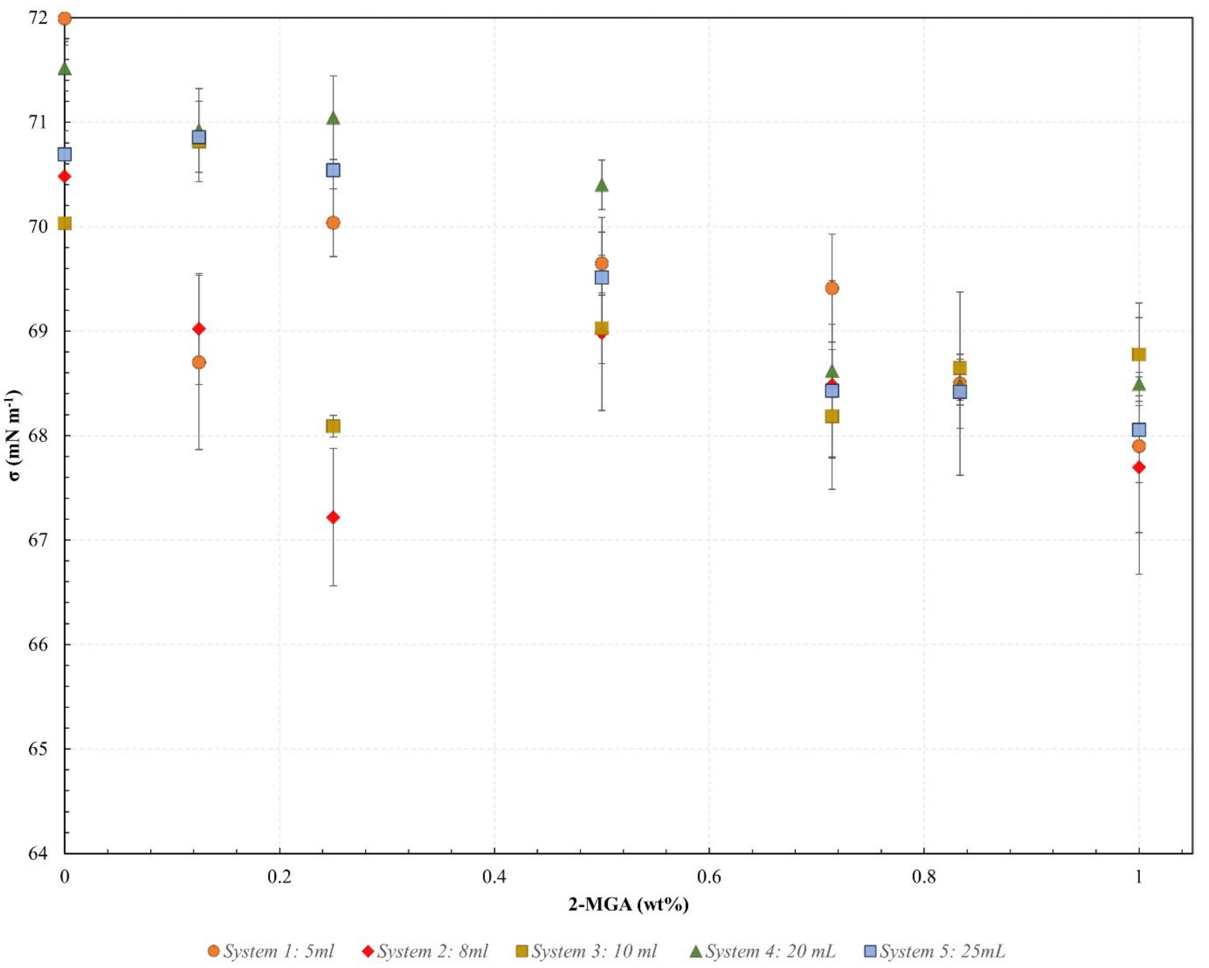

Figure S7. Surface tension values measured for dilute solutions versus weight percent of 2-methylglutaric acid

Table S9. Fractional Surface Area Coverage

2MGA wt%	Average Coverage Probability, φ	Average Surface Tension (mN m^{-1})	Category
5^	0.14	70.94	1
10^	0.30	70.69	1
25	0.41 ± 0.20	69.81 ± 0.40	2
50	0.58 ± 0.17	69.51 ± 0.57	2
60^	0.75	68.92	2
75^	0.94	68.64	3
90^	0.97	68.47	3
100	0.99 ± 0.00	68.28 ± 0.32	3

^Interpolated points

VIII. Summary of experimentally derived GF and *κ* values

Table S10. Experimentally derived GF and κ-values from H-TDMA

Mixture (*wt%*)	GF	$\kappa_{H\text{-}TDMA}$ (Uncorrected)	$\kappa_{H\text{-}TDMA}$ (Corrected)
5% 2-MGA + 95% AS	1.966 ± 0.012	0.612 ± 0.008	0.612 ± 0.008
10% 2-MGA + 90% AS	1.942 ± 0.007	0.604 ± 0.020	0.603 ± 0.020
25% 2-MGA + 75% AS	1.772 ± 0.005	0.608 ± 0.012	0.607 ± 0.012
50% 2-MGA + 50% AS	1.715 ± 0.022	0.567 ± 0.038	0.565 ± 0.038
60% 2-MGA + 40% AS	1.624 ± 0.065	0.453 ± 0.073	0.452 ± 0.072
75% 2-MGA + 25% AS	1.479 ± 0.058	0.316 ± 0.056	0.314 ± 0.055
90% 2-MGA + 10% AS	1.307 ± 0.008	0.172 ± 0.001	0.171 ± 0.001
100% 2MGA	1.151 ± 0.053	0.063 ± 0.024	0.063 ± 0.024

Table S11. Experimentally derived activation diameter and κ-values from CCNC

Mixture (*wt%*)	Activation D_d (nm) (0.4% SS)	Activation D_d (nm) (0.6% SS)	Activation D_d (nm) (0.8% SS)	Activation D_d (nm) (1.0% SS)	κ_{CCNC} (Uncorrected)	κ_{CCNC} (Corrected)
5% 2-MGA + 95% AS	46.2 ± 0.5	36.6 ± 0.0	29.8 ± 0.6	27.2 ± 0.6	0.612 ± 0.043	0.602 ± 0.043
10% 2-MGA + 90% AS	44.6 ± 0.6	36.6 ± 0.0	30.4 ± 0.0	27.1 ± 0.6	0.598 ± 0.032	0.580 ± 0.027
25% 2-MGA + 75% AS	46.5 ± 0.0	36.6 ± 0.0	30.4 ± 0.0	27.3 ± 0.6	0.596 ± 0.034	0.559 ± 0.032
50% 2-MGA + 50% AS	51.9 ± 0.4	40.4 ± 0.4	33.3 ± 0.8	28.7 ± 0.8	0.558 ± 0.031	0.472 ± 0.026
60% 2-MGA + 40% AS[a]	51.2 ± 0.2[a]	41.8 ± 0.3[a]	34.5 ± 0.2[a]	29.9 ± 0.4[a]	0.573 ± 0.027[a]	0.516 ± 0.020
75% 2-MGA + 25% AS	55.8 ± 0.9	44.7 ± 0.6	36.2 ± 0	30.4 ± 0.7	0.401 ± 0.037	0.352 ± 0.025

90% 2-MGA + 10% AS	69.7 ± 1.3	51.8 ± 0.7	35.2 ± 1.5	36.9 ± 0.7	0.235 ± 0.025	0.208 ± 0.023
100% 2MGA	77.6 ± 1.0	60.0 ± 0.5	51.9 ± 1.1	44.7 ± 1.2	0.123 ± 0.010	0.122 ± 0.018

[a] Experimental result from Malek et al. 2023
